# The human TRAM1 locus expresses circular RNAs

**DOI:** 10.1038/s41598-021-01548-0

**Published:** 2021-11-11

**Authors:** Josephine Dubois, Georg Sczakiel

**Affiliations:** 1 Institut für Molekulare Medizin, Universität zu Lübeck and UKSH, Campus Lübeck, Ratzeburger Allee 160, 23538 Lübeck, Germany; 2 Present Address: Division of Hematology and Oncology, Department of Internal Medicine, University of Michigan, Ann Arbor, MI USA

**Keywords:** Cancer, Genetics, Molecular biology, Biomarkers, Molecular medicine

## Abstract

Numerous indirect and in silico produced evidences suggest circular RNAs (circRNA) in mammals while thorough experimental proofs of their existence have rarely been reported. Biological studies of circRNA, however, should be based on experimentally verified circRNAs. Here, we describe the identification of two circRNAs originating from the gene locus of the translocation associated membrane protein 1 (TRAM1). Linear and potentially circular TRAM1-specific transcripts were identified in a transcriptome analysis of urine RNA of bladder cancer (BCa) patients versus healthy donors. Thus, we first focused on the topology of TRAM1-specific transcripts. We describe conclusive experimental evidence for the existence of TRAM1-specific circRNAs in the human BCa cell lines ECV-304 and RT-4. PCR-based methodology followed by cloning and sequencing strongly indicated the circular topology of two TRAM1 RNAs. Further, studies with exon fusion sequence-specific antisense oligonucleotides (asON) and RNase H as well as studies in the use of RNase R contribute to conclusive set of experiments supporting the circular topology of TRAM1 transcripts. On the biological side, TRAM1-specific circRNAs showed low expression levels and minor differences in BCa cell lines while linear TRAM1 transcripts displayed down-regulated expression in the higher cancer stage model ECV-304 versus more differentiated RT-4 cells.

## Introduction

Human bladder cancer (BCa) is one of the most common cancer types worldwide and causes a high number of mortalities^1,2^. The diagnostic gold standard is based on cystoscopy which is invasive, bears a certain health risk, and is related to high costs^3,4^. Alternatively, non-invasive methods like sonography^5^ and urography of the urinary tract^6^ together with microscopic evaluation of urothelial cells in urine^7^ are applied for diagnosis of BCa. Furthermore, cytological evaluation provides specific information about dysplasia of low differentiation but seems to fail to detect low-grade BCa sensitively enough^8^ and is highly dependent on individual expertise of the pathologist^9^. Ultimately, clinical parameters of non-invasive procedures are not sufficient for a single application in primary diagnostics of BCa^4^. Hence, improved biomedical approaches are required for a reliable detection of BCa and for therapy monitoring.

Alternative non-invasive methodology for diagnosis of BCa is based on the analysis of RNA composition in urine of patients. Over the last decades, tumor marker systems increasingly addressed the transcript level, e.g. the Cxbladder Monitor (CxbM) Test^10^ and the XPERT Bladder Cancer Test^11^ on the basis of mRNAs. Further examples also include the metastasis-associated lung adenocarcinoma transcript 1 (MALAT1), a long non-coding RNA (lncRNA)^12^ and diagnostic tests based on urinary micro RNAs (miRNA)^13^. Beside initial diagnostic data the correct classification of BCa is a major goal of future non-invasive approaches. This objective can conceivably be approached by RNA markers for staging and grading of BCa. On a deeper level, more insights are expected from transcriptome analyses of patient RNAs. Examples for transcriptome analyses in the area of BCa already exist for single cells^14^, tissue samples^15^ and urine^16^. Additional independent diagnostic parameters are likely to improve detection and therapy monitoring of BCa because it does not seem to be realistic that cystoscopy will be replaced in the near future^17,18^.

As a new class of potential tumor marker molecules, circular RNAs (circRNA) have been reported to be involved in malignant cell growth^19^. By definition, circRNAs are covalently closed, mostly non-coding transcripts which could be detected for a variety of human genes^20–22^. Further, circRNA species are generated in a non-canonical fashion during alternative splicing of transcripts which leads to a covalent bond of 5’- to 3’- ends for exonic circRNAs^23^. This linkage is termed back splice junction (BSJ) and forms the characteristic sequence of circular transcripts. Mechanistically, the generation of circRNAs can be supported by flanking introns via base pairing of inverse complementary sequences^23^. Moreover, studies on human cell lines indicated an independent regulation of circRNA expression from their respective linear transcripts^24^.

Owing to the absence of free ends, circRNAs are believed to exist at higher half-life in cells compared to linear transcripts. While 10 h was estimated for the half-life of mRNAs^25^, the average half-life measured for circRNAs was approximately 48 h^20^. This different endogenous stability seems to be due to the resistance of circular transcripts against nucleases like RNase R and other cellular exonucleases^20^. Therefore, circRNAs appear to be more easily detectable in many tissues and liquid biopsies^21,26^. There is also increasing evidence for circular transcripts in serum^27^, saliva^28^, and urine^29^. This suggests a high potential of circRNAs as biomarkers for a non-invasive detection in tumor diagnostics. Further, packaging into exosomes can increase the stability of circRNAs in liquid biopsies and may function as nuclease-resistant environment for transportation to distant tissues and organs^27^.

Another important aspect in the context of cancer detection and monitoring is related to the tissue- and development-specific expression of circular RNA species^30^. For example, a deregulation of circRNAs in tumors was reported as was a correlation with clinicopathological parameters like staging and size of tumors^31^. An example for BCa is circRNA-MYLK which displays a significant overexpression. The circRNA species was positively correlated with vascular endothelial growth factor A (VEGFA) mRNA. This is thought to be related to an increase in cell proliferation and migration, thereby providing a link of staging and grading of BCa to the expression level of circRNA-MYLK^32^. Further, circHIPK3 and circCDYL could be identified as promising target molecules for an early screening of BCa which is a major goal of non-invasive tumor diagnostics^33^. To reach this goal, urine seems to be a suitable source for biomarkers as direct physical contact between tumor cells and urine seems to be given. In addition, gene expression of circRNAs was detected in urine by next generation sequencing of exosomal RNA in prostate carcinoma patients^34^ and via microarray analysis of free circRNAs in BCa patients^29^. These results suggest an important role for circular transcripts in diagnosis, monitoring, and treatment of cancer^35,36^.

It is important to note that this study focuses on the biogenesis and characterization of circular RNA derived from the translocation associated membrane protein 1 (TRAM1) gene locus which was identified by transcriptome analyses in the use of urine RNA from patients of bladder cancer versus healthy donors^37^. Hence, we first summarize the clinical background of TRAM1 and, subsequently, the molecular characterization of transcripts of TRAM1 and in particular of circular variants. The experimental evidence of TRAM1 transcripts included PCR-based detection in the bladder cancer cell lines ECV-304 and RT-4 and analysis of differential gene expression. This was completed by cloning and sequencing of developed amplicons, RNase H-based studies and a deep characterization of circular topology of TRAM1 specific RNAs.

## Results

### Transcriptome analyses of urine RNA shows decreased expression of TRAM1 in bladder cancer patients

In order to study RNA-based markers for non-invasive diagnosis of bladder carcinoma (BCa), we performed a systematic, whole transcriptome analysis of urine RNA collected from healthy donors (C) and patients with high risk of BCa (HR) as described recently^37^. Briefly, this recent work included acquisition of urine, stabilization of urine RNA, delivery and storage of samples, and RNA preparation from whole urine^38–40^. Next, seven samples representing the mean values of markers determined recently in a large set of urine samples were pooled and used to build the final patient pools C and HR (Supplementary Table S1). Both urine RNA pools were used to produce cDNA libraries followed by deep transcriptome analyses.

**Figure 1:**
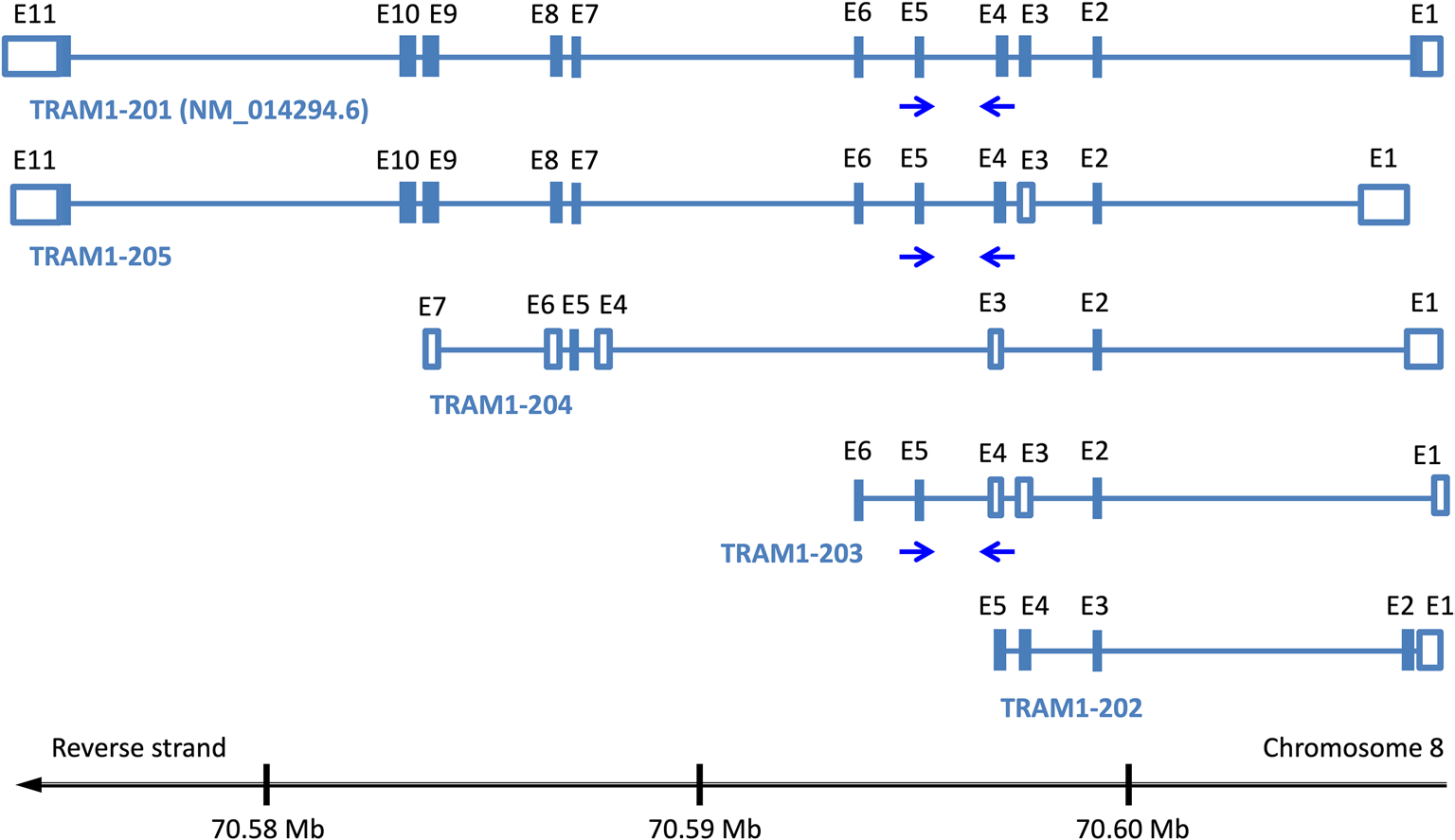
Linear transcripts of the TRAM1 gene locus. Five linear RNA species are predicted for the TRAM1 gene locus by the Ensembl database. These transcripts are located on the reverse strand of chromosome 8 (black line, lower panel). Exact locations of TRAM1 transcripts on chromosome 8 are shown in Table 1. Coding exons are indicated by blue boxes, non-coding exon sequences are shown as open boxes. Introns are represented by blue lines. Exons are numbered from E1 to E11 with letters in black. Blue arrows indicate the convergent primer pair which can bind to TRAM1-201, TRAM1-205 and TRAM1-203.

To identify differentially expressed transcripts between the C group and the HR group of BCa patients, we first ranked urine RNAs identified by whole transcriptome analyses according to lg_10_(fold change), abbreviated lg_10_(fc) values comparing both urine pools. Further, we defined bioinformatic, molecular, and biological criteria for selection of promising marker candidates^37^. Finally, linear and circular transcripts of the TRAM1 gene locus were listed as marker candidates as they show an obvious correlation with the health status of donors (Table 1). Two linear TRAM1-specific RNA species (TRAM1-205, TRAM1-201) show a slightly decreased expression in pooled urine RNA of HR patients compared to healthy donors. Moreover, TRAM1-203 indicates a strong decrease in the HR group with a lg_10_(fc) value of − 1.09. We therefore analyzed the structure of all primary linear transcripts predicted for the TRAM1 gene locus by Ensembl (Fig. 1). This enabled the design of convergent primer pairs for the amplification of linear sequence segments and included only TRAM1 RNA species with negative lg_10_(fc) values. We would like to note that the TSL value of TRAM1-203 is related to 5 and the existence of this transcript seems to be uncertain (Table 1). On the other hand, all circular TRAM1 transcripts show reduced presence in the HR group compared to the control group. Hence, divergent primer pairs for the specific detection of circular transcripts were developed to target all circular TRAM1 splicing variants resulting in a lg_10_(fc) value of − 1.26. In summary, transcripts of the TRAM1 gene locus represented the most promising candidates for potent tumor markers with decreased gene expression in HR patients.

**Table 1:** Linear and circular transcripts of translocation associated membrane protein 1 (TRAM1).

Transcript ID	Name	Location	Exons	Length [b]	HR [TPM]	C [TPM]	lg_10_(HR/C)	TSL	Biotype
**ENST00000520700**	**TRAM1-203**	**chr8: 70,594,501–70,608,352**	**6**	**542**	**1.63**	**20.12**	− **1.09**	**5**	**Pt**
**ENST00000521425**	**TRAM1-205**	**chr8: 70,573,442–70,607,778**	**11**	**3394**	**5.13**	**8.14**	− **0.20**	**2**	**Pc**
**ENST00000262213**	**TRAM1-201**	**chr8: 70,573,218–70,608,416**	**11**	**3056**	**6.08**	**8.43**	− **0.14**	**1**	**Pc**
ENST00000521049	TRAM1-204	**chr8: 70,583,706–70,608,339**	7	872	3.70	2.81	0.12	5	Pt
ENST00000518678	TRAM1-202	**chr8: 70,597,891–70,608,387**	5	535	4.34	3.04	0.15	4	Pc
**Mean values**	–	–	–	–	**4.28**	**12.23**	–	–	–
**Final lg**_**10**_**(fc) value**	–	–	–	–	–	–	− **0.46**	**–**	**–**

Transcript ID	Name	Location	Exons	Length [b]	HR [TPM]	C [TPM]	lg_10_(HR/C)		
**hsa_circ_0084758**	**circTRAM1-58**	**chr8: 71,508,497–71,510,246**	**2**	**176**	**0.66**	**41.33**	− **1.80**		
**hsa_circ_0084756**	**circTRAM1-56**	**chr8: 71,506,740–71,510,490**	**4**	**383**	**0.81**	**21.56**	− **1.43**		
**hsa_circ_0084757**	**circTRAM1-57**	**chr8: 71,506,740–71,512,317**	**5**	**447**	**0.98**	**18.51**	− **1.28**		
**hsa_circ_0084759**	**circTRAM1-59**	**chr8: 71,508,497–71,520,371**	**5**	**422**	**3.08**	**20.04**	− **0.81**		
**Mean values**	–	–	–	–	**1.38**	**25.36**	–		
**Final lg**_**10**_**(fc) value**	–	–	–	–	–	–	− **1.26**		

All TRAM1 RNA species detected in the transcriptome data from our previous study^37^ are listed. Further, we list transcripts per kilobase million (TPM) and transcript support levels (TSL). TSL provided by Ensembl gives insights into the real existence of bioinformatically predicted transcripts. Transcripts indicated by bold letters are targeted by the convergent primer pair and were used for calculation of mean TPM values for the HR and C patient group^37^. The mean TPM values result in a finally calculated lg_10_(fold change) value for a potential new biomarker. (Legend: Protein coding (Pc), Processed transcript (Pt)).

**Figure 2:**
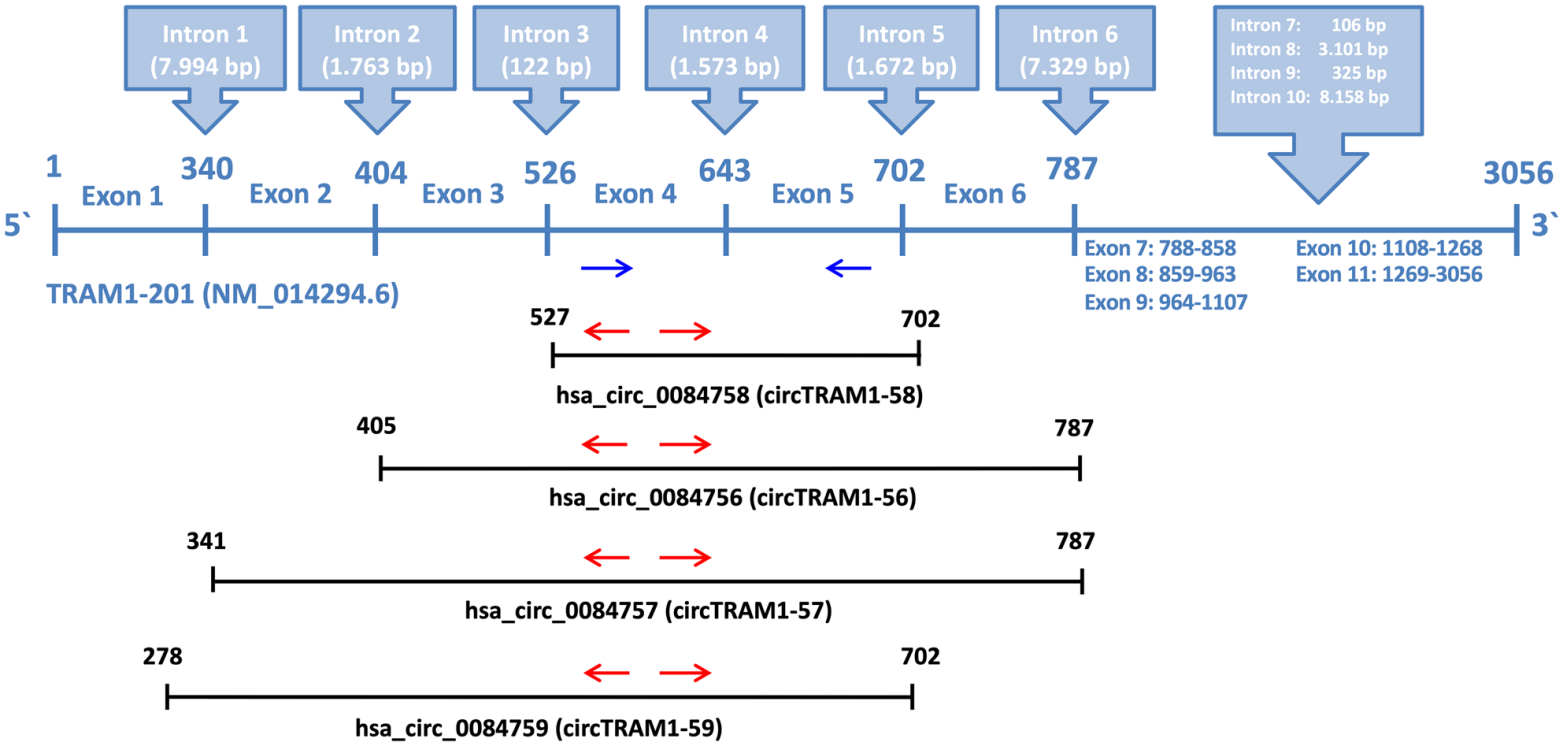
Schematic representation of linear and circular TRAM1-specific transcripts. The exons of the primary transcript TRAM1-201 are shown as a blue line (middle panel). Exons are numbered from 1 to 11 and the positions of the introns (blue boxes, upper panel) are indicated by white numbers in bold. The combined exons that produce circRNA after back-splicing are indicated by lines in black (lower panel). Blue arrows indicate the convergent primer pair which can bind to linear and circular TRAM1 transcripts and could detect all TRAM1 RNA species shown in this figure. Red arrows indicate divergent primer pairs detecting only TRAM1 circRNAs. Names and positions of the TRAM1-precursors of circRNAs are indicated at the lower panel.

**Figure 3:**
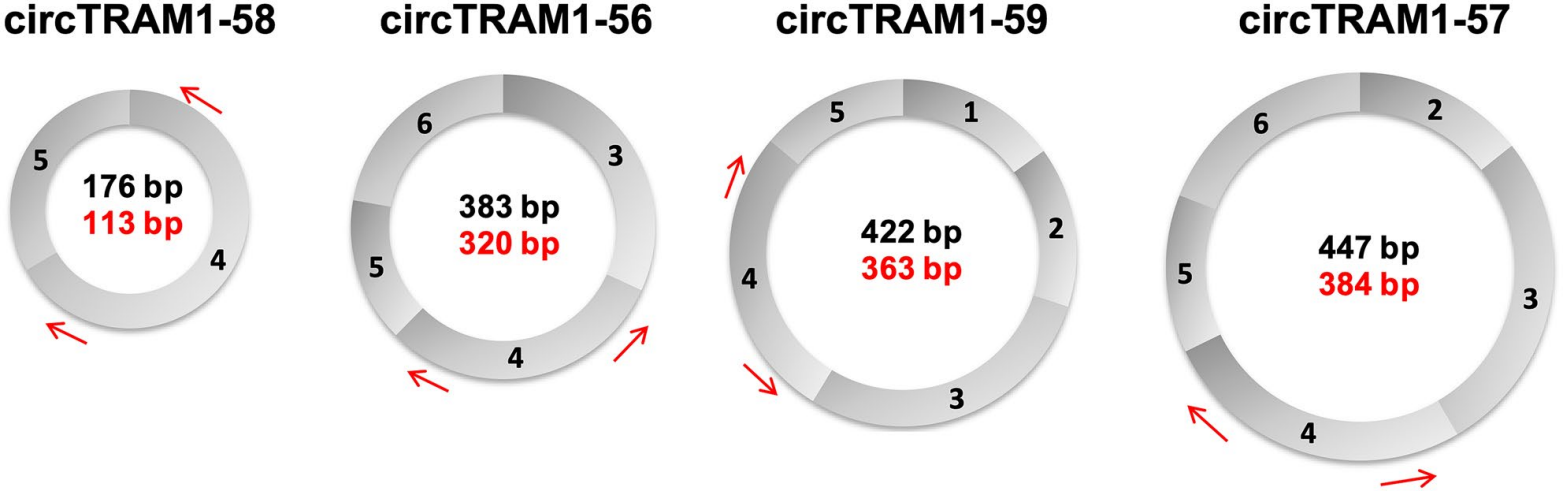
Circular transcripts of the TRAM1 gene locus. Red arrows indicate divergent primer pairs detecting only circular TRAM1 transcripts. The length of mature circRNA is shown in black and the length of PCR amplicons for each circRNA is shown in red. Names of TRAM1 circRNAs are indicated in the upper panel and exons of each circRNA species are indicated by numbers.

The linear transcript TRAM1-201 consists of 11 exons and it is the template for generation of all circRNAs of the TRAM1 gene locus according to circBase (Fig. 2). Further, exons 1–6 are involved in the formation of circRNAs and exons 4 and 5 are part of all TRAM1 circRNAs (Figs. 2, 3). Hence, the convergent primer pair to detect linear and circular TRAM1 transcripts was positioned in exons 4 and 5. Divergent primer pairs were designed according to the circRNA with the lowest lg_10_(fc) value, circTRAM1-58, and placed in exon 4.

### Detection of TRAM1 transcripts by PCR

**Figure 4:**
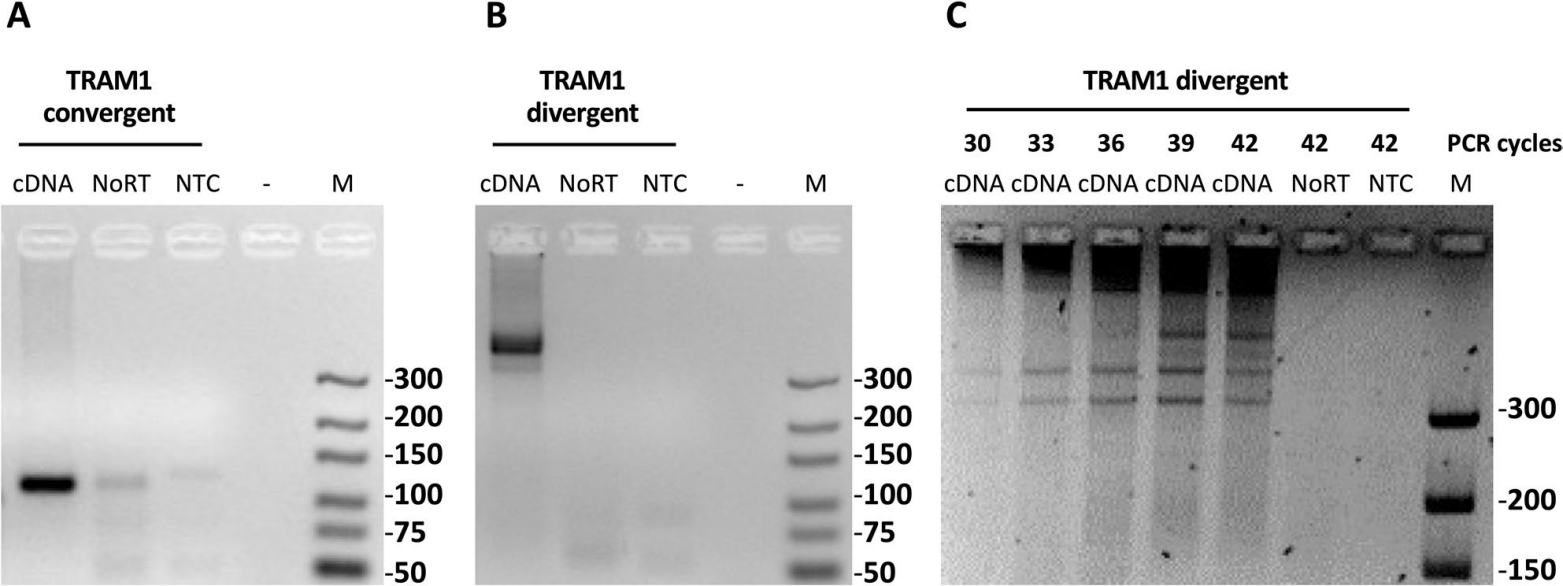
PCR-based detection of linear and circular TRAM1 transcripts. 250 ng cDNA derived from RNA from ECV-304 cells were amplified by 40 PCR cycles (**A**, **B**) or by the numbers of cycles indicated in panel, upper part (**C**). PCR products were separated by 5% agarose gels. (**A**) Use of the convergent primer pair which amplifies the calculated amplicon length of 111 bp for linear TRAM1 transcript fragments. (**B**) Amplification of circular TRAM1 transcripts using the divergent primer pair showed PCR signals > 300 bp. (**C**) Amplification products of the divergent primer pair as a function of PCR cycles (indicated at the upper part). Full-length gels are shown in Supplementary Figure S1.

The source of RNA for the detection of TRAM1 transcripts by PCR was ECV-304 cells which had been derived from a G3 bladder carcinoma^41,42^. Subsequent to cDNA synthesis, PCR was performed and products were analysed by 5% agarose gels (Fig. 4). PCR using the convergent primer pair which amplifies linear transcript fragments, resulted in the expected amplicon length for TRAM1 transcripts (Fig. 4A). Circular TRAM1 transcripts are detected with divergent primer pairs (bands > 300 bp, Fig. 4B) which we assigned to circTRAM1-56, circTRAM1-59, and circTRAM1-57 (Figs. 3, 4B). It should be noted that we did not find evidence for circTRAM1-58 in ECV-304 cells. In order to gain more information about PCR products of the divergent primer pair, different numbers of PCR cycles were run (Fig. 4C). Visualization of the PCR products led to two distinct bands visible after 30, 33, and 36 cycles, and even more distinct PCR products after more PCR cycles. In conclusion, increasing numbers of PCR cycles resulted in a greater number of specific products at varying band intensity. While we cannot unequivocally relate bands to the calculated length of TRAM1-specific circRNAs (Fig. 3), at least two hypotheses might explain the observed pattern of PCR products. Firstly, these bands may represent the three TRAM1 circRNAs mentioned before (Fig. 4B) or amplification products of sequence segments of a concatemer generated during reverse transcription of circRNAs by a rolling circle mechanism.

### Cloning and sequencing of TRAM1 transcripts

Next we tried to get definitive sequence information by cloning and sequencing of PCR products generated by TRAM1-specific primer pairs. Briefly, PCR products produced by convergent and divergent primer pairs were ligated into pCRII vector and transformed into *E. coli* DH5$\mathrm{\alpha }$ bacteria cells. Single clones were expanded and plasmid DNA was prepared for analyses by sequencing.

**Figure 5:**
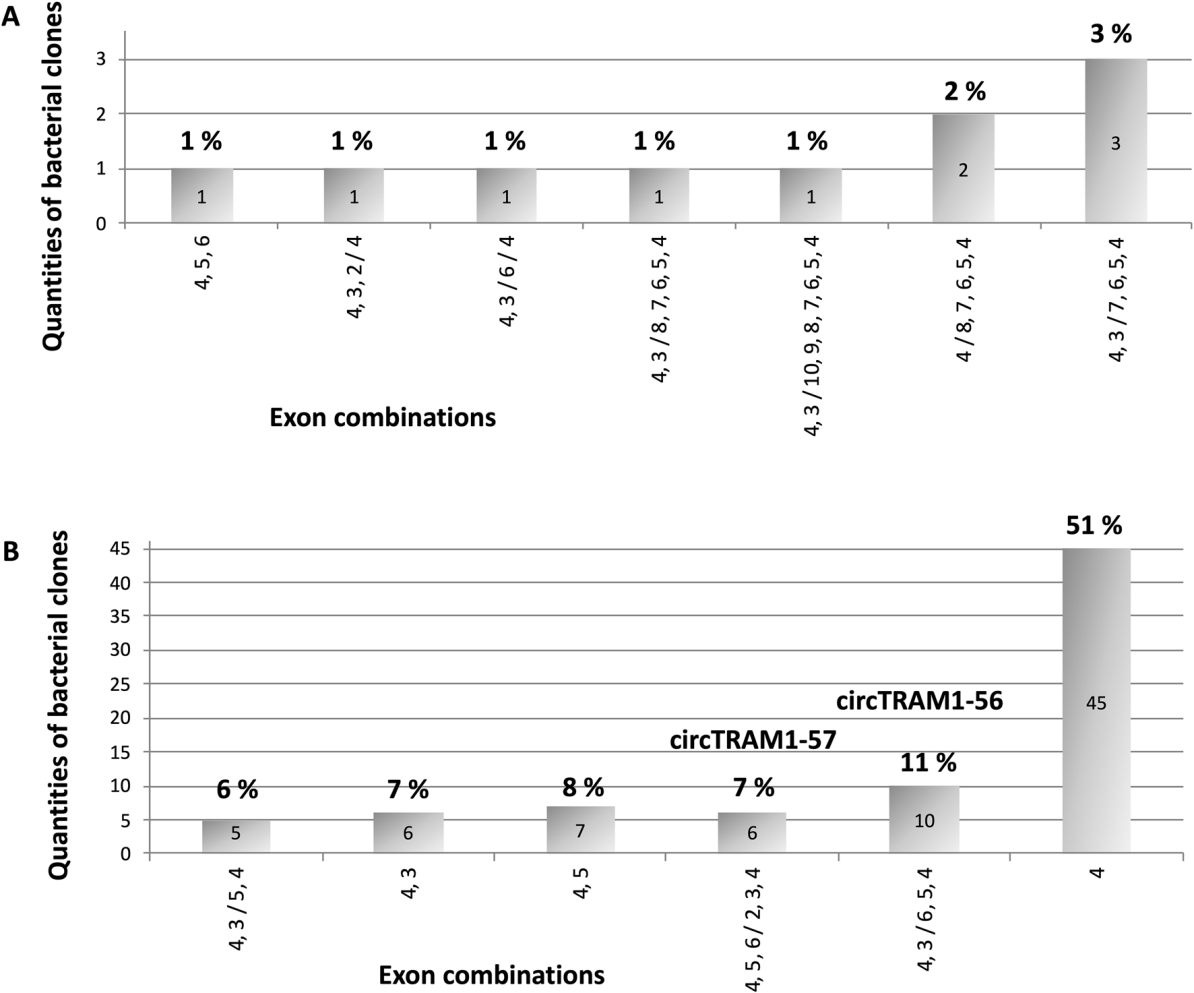
Composition of cloned TRAM1-specific PCR products. PCR amplicons of the divergent primer pair were cloned into pCRII vector of *E. coli* DH5$\mathrm{\alpha }$ and sequences were determined. The quantity of identified TRAM1 fragments is indicated within the bars. Percentages and identified circular TRAM1 transcripts are indicated on top of the bars. Individual exons and their order are shown below the X-axis. Exon numbers are combined by commas and ordered as present in linear transcripts. The slash indicates a potential circular back-splice junction. (**A**) Summary of less abundant potential circular TRAM1 transcripts. (**B**) Summary of frequent potential circular TRAM1 transcripts.

For PCR product of the convergent primer pair, the correct TRAM1 sequence of linear transcript fragments was confirmed. Amplification using the divergent primer pair resulted in a variety of different TRAM1-specific sequences of various lengths. Figure 5 summarizes the composition of less abundant TRAM1-sequences equal to or below 3% frequency of all clones (A) and more abundant sequences at or above 6% frequency (B).

This cloning-based analysis of the composition and relative amounts of TRAM1-specific potentially circular RNAs indicates that less abundant potential TRAM1 transcripts (Fig. 5A) mainly consist of longer TRAM1 sequences. We would like to note that PCR conditions of this experiment were chosen in favor of shorter amplification products. We interpret the results such that exon combination 4,5,6 indicates linear TRAM1-specific sequences and exon order 4,3/6/4 seems to be not an authentic TRAM1 transcript because exon 5 is missing. All other TRAM1 sequences represent possible circular transcripts. In addition, most potential back splice junctions (BSJ) occurred at exon 3. However, it does not seem to provide reliable insights to study these potential circular TRAM1 transcripts as they constitute only a small fraction of all detected sequences. The majority of cloned TRAM1 sequences (Fig. 5B) contain linear TRAM1 sequences like 4,3, 4,5 and 4 and circular sequences such as 4,3/5,4, 4,5,6/2,3,4, and 4,3/6,5,4, respectively. The latter three exon combinations were analyzed in detail concerning their exon order, indication of artifacts, and correct BSJs. These three criteria matched to most of TRAM1 sequences assigned to circTRAM1-56 and circTRAM1-57, i.e. these two circRNAs are validated in this approach. Furthermore, both circular transcripts constitute approximately 10% of all TRAM1 transcripts and, thus, could play an important biological role. On the other hand, the potential circRNA 4,3/5,4 is not one of the predicted transcripts. However, this order of exons could indicate a potential circRNA. A deeper look into sequence data revealed minor deviations from the predicted exon sequences as well as primer duplications and inconsistent BSJs. Consequently, this exon combination, i.e. circRNA 4,3/5,4 was excluded from further studies. In summary, these findings stress the requirement of detailed analyses of the exon structure and BSJs of identified sequences.

In a next step, distinct PCR bands of the divergent primer pair (Fig. 4B) were isolated by agarose gel electrophoresis and gel elution. Cloned fragments were sequenced as described. This experiment confirmed the sequence of circTRAM1-56. Thus, novel circular TRAM1 transcripts have been reliably identified, i.e. circTRAM1-56 and circTRAM1-57.

### RNase H-based studies identify circular TRAM1 transcripts

**Figure 6:**
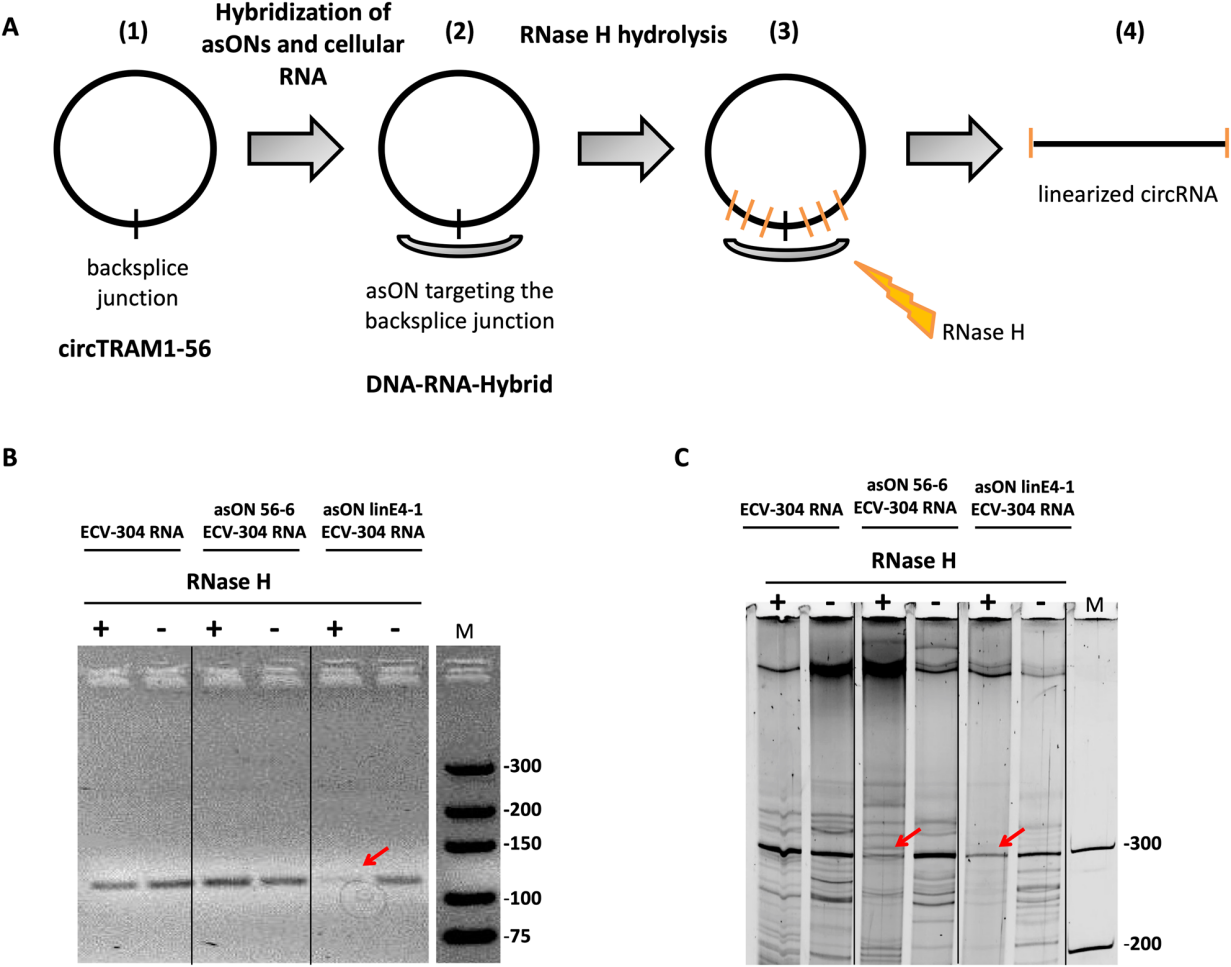
RNase H treatment of complexes between cellular RNA and TRAM1-specific asON indicate circular topology. (**A**) Schematic illustration of the experimental setup. (1) TRAM1 circRNAs are present in ECV-304 cellular RNA. (2) Antisense oligonucleotides (asON) are directed against back-splice junctions of circRNAs. (3) The RNA portion of DNA-RNA hybrids is cleaved by RNase H. (4) circRNA is converted to linear strands which cannot be amplified by PCR with the divergent primer pair. (**B**, **C**) PCR-based gel analysis of RNase H treatment. ECV-304 RNA was hybridized with different asONs targeting circTRAM1-56 (asON 56–6) or linear and circular TRAM1 RNAs (asON linE4-1). For details see Suppl. Table S2. Samples incubated with RNase H (+) or without enzyme (−) are indicated on top of each lane. Products of conventional PCR using the convergent primer pair (**B**) showed the expected PCR signals at 111 bp and were analyzed by 5% agarose gels. PCR products of the divergent primer pair (**C**) were separated by 10% denaturing PAGE and displayed the expected signal at 300 bp. Full-length gels are shown in Supplementary Figure S2.

In order to provide another independent experimental clue of the circular topology of TRAM1 transcripts we developed an approach based on RNase H-mediated cleavage and product analysis. Antisense oligonucleotides (asONs) spanning the back-spliced fusion region were incubated with RNA preparations followed by RNase H treatment. Cleavage indirectly monitors the circular structure of treated RNAs (Fig. 6A). Briefly, asONs were designed to target circTRAM1-56 or all TRAM1 transcripts. The asON targeting linear and circular TRAM1 transcripts was designed to bind to exon 4 as this is part of all TRAM1 RNA species. Moreover, the asON to specifically hybridize to circTRAM1-56 was designed according to its BSJ sequence. After hybridization of asONs with cellular RNA prepared from ECV-304 cells and treatment with RNase H, RNA products were converted to cDNA by reverse transcription followed by PCR using the convergent primer pair (Fig. 6B) or the divergent primer pair (Fig. 6C).

The data shown in Fig. 6 indicate the presence of circTRAM1-56 RNA in ECV-304 cells. Firstly, asON targeting linear and circular TRAM1 transcripts (asON linE4-1, Fig. 6B, C) indicated a decrease of band intensity after RNase H treatment suggesting that asON binding to exon 4 of TRAM1 transcripts occurred. Further, the negative control, ECV-304 RNA alone, showed no signal reduction (ECV-304 RNA, Fig. 6B, C) in presence or absence of RNase H, i.e. it remained intact rather than being degraded by the enzyme. Lastly, ECV-304 RNA displayed no decrease of band intensity after hybridization with the asON targeting circTRAM1-56 and the use of the convergent primer pair (asON 56-6, Fig. 6B). This primer pair mainly amplifies linear TRAM1 RNA species as the circRNAs only represent a small fraction of TRAM1 transcripts. In consequence, we would not expect to detect a distinct reduction of PCR signal of the convergent primer pair. Conversely, the asON targeting circTRAM1-56 indicated a substantial decrease of band intensity after RNase H treatment and amplification with the divergent primer pair (asON 56-6, Fig. 6C). In conclusion, positioning of an asON against the BSJ sequence led to specific targeting of circTRAM1-56 without affecting linear TRAM1 transcripts. Moreover, RNase H action on circular RNAs is possible and the whole experimental setup was functional on the RNA level. This approach could therefore be further applied on functional knockdown studies in cells using modified asONs. Finally, the PCR bands of circTRAM1-56 validated via cloning and sequencing could be also confirmed directly on the RNA level and gave further hints on the real existence of this circular RNA species in cells.

### Analyses of circTRAM1-56 and circTRAM1-57

**Figure 7:**
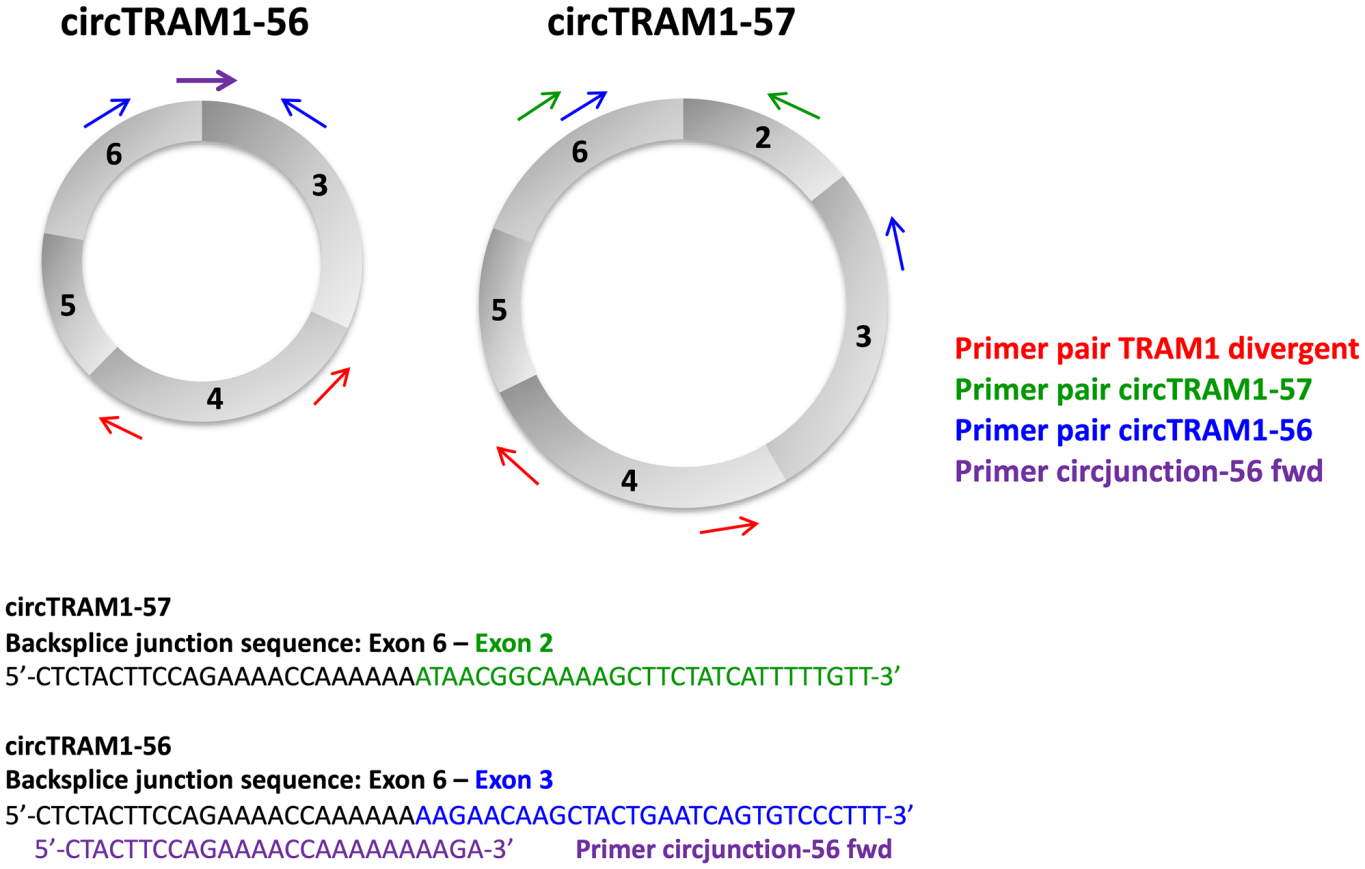
Positioning of PCR primer for the detection of circTRAM1-56 and circTRAM1-57. Schematic illustration of both circular TRAM1 transcripts and the divergent primer pair targeting all TRAM1 transcripts (red arrows). Green arrows indicate primer for specific detection of circTRAM1-57 which were positioned in exon 2 and exon 6. The corresponding back-splice junction (BSJ) sequence is shown in the lower panel where nucleotides of exon 6 are labelled by black color and bases of exon 2 are marked in green. The divergent primer pair for amplification of circTRAM1-56 was first positioned in exon 6 and exon 3 (blue arrows) but also lead to detection of circTRAM1-57. For the specific amplification of circTRAM1-56, the new forward primer (purple arrow) was positioned directly on the BSJ of the circRNA. The corresponding BSJ sequence is shown in in the lower panel where bases of exon 6 are indicated by black color and bases of exon 3 are labelled in blue. The binding position of the new forward primer (circjunction-56 fwd) is indicated by purple color.

For both circular RNA species, we designed divergent primer pairs recognizing the two confirmed transcripts circTRAM1-56 and circTRAM1-57. The binding sites for primer along both circRNAs are depicted in Fig. 7. In case of circTRAM1-57 the forward primer binds within exon 6 and the reverse primer binds within exon 2. The resulting PCR product was specific for circTRAM1-57 and was confirmed by sequencing. The specific detection of circTRAM1-56 was more complex. Positioning of primer in exon 3 and exon 6 lead to amplification of both circular TRAM1 RNA species (Fig. 7) which is not useful for quantification of single cellular transcripts. Therefore, we modified the protocol and developed an alternative positioning of forward primer directly on the BSJ of circTRAM1-56, because this sequence is the only difference between both circular TRAM1 RNA species. Moreover, the new forward primer had to be specific for exon 3 as the BSJs of circTRAM1-56 and circTRAM1-57 share exon 6 in their BSJ sequences. Further, detection of linear TRAM1 transcripts should be avoided. To overcome these hurdles, the new forward primer was positioned 4 nucleotides within exon 3 as the 3’-end defines specificity of Taq polymerases. This strategy led to a specific PCR signal for circTRAM1-56 without detecting circTRAM1-57 and was also confirmed by sequencing. Full length sequences of spliced transcripts and PCR amplicons for circTRAM1-56 and circTRAM1-57 are provided in Supplementary Table S2.

### Sequence context and circular topology of TRAM1 specific transcripts

#### Analysis of flanking noncoding sequences of circular TRAM1 transcripts

The analysis of flanking introns of potential circular transcripts could shed light on the probability of the biogenesis of these RNA species in cells. Hence, the predicted TRAM1 circRNAs were examined for inverse complementary sequences of their flanking noncoding regions (Table 2).

**Table 2:** Analysis of flanking noncoding sequences of circular TRAM1 transcripts.

Transcript	Involved intron	Inverse complementary sequences
circTRAM1-58	intron 3, intron 5	No inverse complementary sequences
circTRAM1-56	intron 2, intron 6	334 nts with 67.4% sequence match 351 nts with 65.2% sequence match 465 nts with 61.9% sequence match 8 further alignments of 74–188 nts with 58.7–70.7% sequence match
circTRAM1-57	intron 1, intron 6	389 nts with 70.2% sequence match 346 nts with 68.8% sequence match 375 nts with 68.3% sequence match 35 further alignments of 64–464 nts with 57.3–76.6% sequence match
circTRAM1-59	–, intron 5	No inverse complementary sequences

Noncoding sequences were obtained from Ensembl by analyzing the respective linear reference transcript. Flanking introns of all predicted circular TRAM1 transcripts were examined concerning inverse complementary sequences. Local alignments were performed and the three best sequence matches are shown with quantities and percentages of complementary nucleotides (nts). Detailed analysis of intron sequences for circTRAM1-56 and circTRAM1-57 is presented in Supplementary Tables S3 and S4.

For the predicted circular TRAM1 RNA species circTRAM1-58 and circTRAM1-59 we could not identify inverse complementary sequences which is compatible with the findings of cloning experiments. Therefore, the existence of both circular transcripts in cells seemed to be unlikely. In contrast, for the PCR-confirmed transcripts circTRAM1-56 and circTRAM1-57 inverse complementary intron sequences with lengths of 334–465 nucleotides and sequence matches up to 70% were found. Hence, the introns 1, 2, and 6 could be involved in the generation of the detected circular TRAM1 transcripts via base pairing of inverse complementary sequences by a process that could involve a spatial proximity of exon ends and therefore might facilitate the biogenesis of back-splicing in cells. However, we would like to note that this is no direct experimental evidence.

#### Examination of tandem repeat hypothesis for circular TRAM1 transcripts

**Figure 8:**
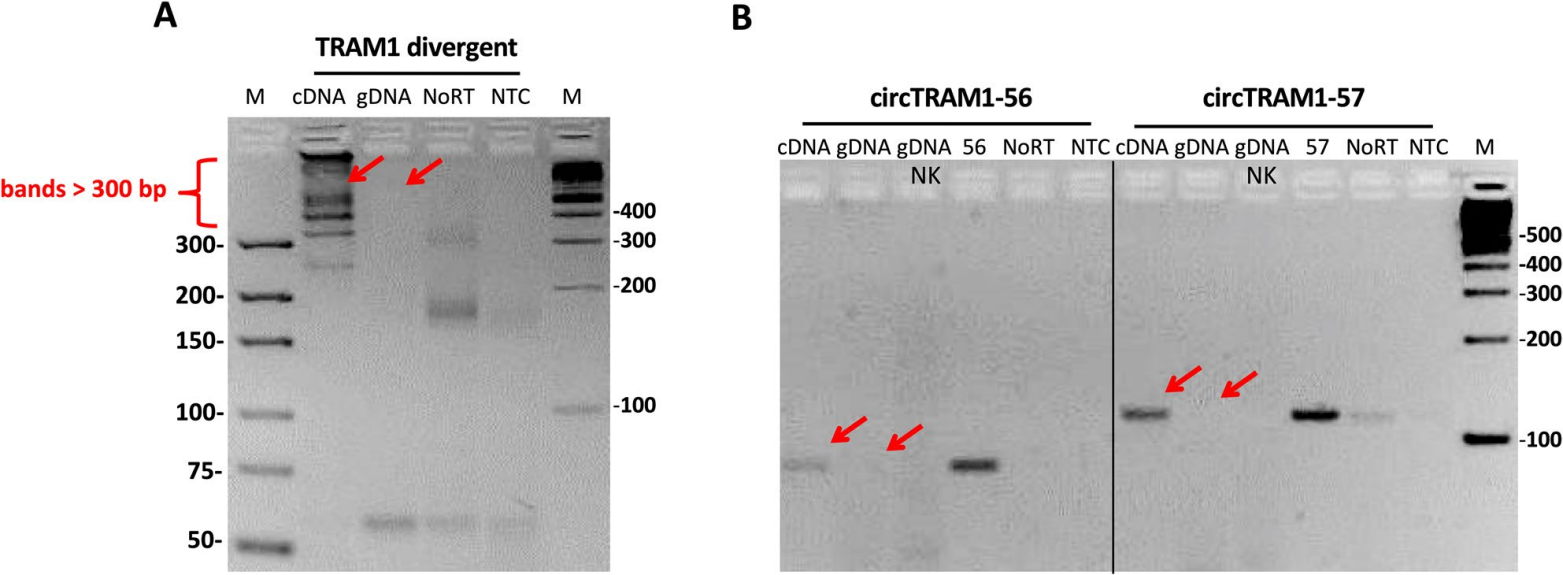
Examination of the tandem repeat hypothesis for circular TRAM1 transcripts. The tandem repeat hypothesis was tested with ECV-304 DNA (gDNA) and compared with cDNA amplicons of the detection of different TRAM1 circRNAs. (**A**) 250 ng cDNA or gDNA were amplified by conventional PCR using the divergent primer pair for detection of all predicted TRAM1 circRNAs. (**B**) 50 ng cDNA or gDNA were amplified using SYBR Select Master Mix for detection circTRAM1-56 and circTRAM1-57. Negative controls and 800 pg of plasmids carrying the sequence of circTRAM1-56 (“56”) or circTRAM1-57 (“57”) were included in the PCR reaction. The sample “gDNA NK” describes a negative control consisting of only ECV-304 gDNA and water. Red arrows indicate the correct cDNA amplicons of each PCR reaction and the missing PCR signal of amplification of ECV-304 gDNA. Samples were analyzed in 3% agarose gels. Full-length gels are shown in Supplementary Figure S3.

The tandem repeat hypothesis describes the existence of exon duplications in the genome of cells which lead to false positive detection of seeming BSJs in PCR reactions using the divergent primer pair^43^. In order to exclude this possibility, genomic DNA of ECV-304 cells was used to test all divergent primer pairs detecting different TRAM1 circRNAs and possible tandem repeats on the DNA level (Fig. 8).

The PCR reactions of ECV-304 cDNA using the divergent primer pair for the detection of all predicted TRAM1 circRNAs (Fig. 8A), circTRAM1-56 or circTRAM1-57 (Fig. 8B) showed the expected PCR signals. Conversely, in ECV-304 chromosomal DNA we found no evidence for PCR amplification with divergent primer pairs. We conclude that the amplicons of divergent primers have no origin on the DNA level and are not the result of exon repeats in ECV-304 DNA. In summary, the tandem repeat hypothesis was excluded as a source for false positive detection of seeming BSJs.

#### RNase R treatment of TRAM1 transcripts

In this study, we finally examined the circular topology of TRAM1 circRNAs by RNase R hydrolysis which leads to an efficient reduction of linear transcripts whereas circular RNA species are not targeted by the enzyme^44^. For this purpose, ECV-304 RNA was treated with RNase R and compared with untreated cellular RNA. TRAM1 transcripts were quantified in both samples via qPCR using the previously developed convergent and divergent primer pairs (Table 3).

**Table 3:** RNase R hydrolysis of linear and circular TRAM1 transcripts.

Transcript	A) copies cDNA − RNase R	B) copies cDNA + RNase R	lg_10_(B/A)
TRAM1	66,353.49	223.56	− 2.47
circTRAM1-56	16.18 13.66	86.13 74.90	0.73 0.74
circTRAM1-57	100.04 94.22	144.75 105.11	0.16 0.05

Quantification of RNase R-treated and untreated ECV-304 cellular RNA via qPCR using convergent and divergent primer pairs to target TRAM1 transcripts. lg_10_(fold change) values were calculated from copies of each PCR amplicon in both samples to compare different transcript types.

In this experiment, TRAM1 transcripts amplified with the convergent primer pair, which mainly amplify linear RNA species, show a clear reduction of more than two orders of magnitude in ECV-304 RNA treated with RNase R. In contrast, quantification of circTRAM1-56 and circTRAM1-57 indicated similar or slightly increased copies after RNase R treatment. The effect of a minor increase of amplification products after treatment with RNase R is surprising. However, this effect was also observed by others^45^. We further note that a normalization of samples based on a linear standard transcript is not possible which could explain the slight increase in copy numbers. This RNase R-based experiment is compatible with the amplification of TRAM1 circRNAs in PCR reactions using divergent primer pairs. Finally, we listed a set of experimental evidences for the existence of both circular TRAM1 transcripts, circTRAM1-56 and circTRAM1-57, in ECV-304 cells.

### Analysis of differential gene expression of TRAM1 transcripts in urinary bladder carcinoma cell lines

The analysis of differential gene expression of linear and circular TRAM1 transcripts was performed to verify the results of transcriptome data of urine RNA from healthy donors (C) and HR patients of BCa. Due to limited amounts of RNA in human urine and the lack of sufficient total patient material, we performed initial technical studies in the use of a cell culture model system which was described recently^39^. In this dual cell model, the human control group C is represented by RT-4 cells which were established from a G1 stage urinary bladder carcinoma^46^. The human HR group is reflected by ECV-304 cells which had been derived from a G3 bladder carcinoma^41,42^. Thus, we validated PCR amplicons of linear and circular TRAM1 transcripts in both cell lines and compared calculated lg_10_(fc) values with the data of transcripts in urine samples of patients (Table 4).

**Table 4:** Different gene expression of linear and circular TRAM1 transcripts in ECV-304 and RT-4 cells and in urine RNA of C and HR groups.

Transcript	HR [TPM]	C [TPM]	lg_10_(HR/C)	ECV-304	RT-4	lg_10_(ECV-304/ RT-4)
TRAM1	4.28	12.23	− 0.46	3287.14	12,809.89	− 0.59
circTRAM1-56	0.81	21.56	− 1.43	9.96	17.56	− 0.25
circTRAM1-57	0.98	18.51	− 1.28	87.81	187.96	− 0.33

TPM and lg_10_(fold change) values are listed for HR and C groups and for the cell culture models RT-4 and ECV-304. TRAM1 transcripts in bladder cancer cell lines were measured by RT-qPCR using convergent and divergent primer pairs. All data are normalized to values of 18S rRNA and used for calculation of lg_10_(fold change) values. Unpaired *t* test revealed statistical significance for differential gene expression of BCa cell lines (*P* ≤ 0.05).

For TRAM1 transcripts, lg_10_(fc) values are consistent between pooled urine RNA samples of C and HR group and bladder cancer cell lines. The linear TRAM1 RNA species show decreased expression in urine of HR BCa patients and the G3 model ECV-304. In contrast, the strong under-expression of both circular TRAM1 transcripts in HR patients was not consistent with ECV-304 cells in which only a minor reduction of circTRAM1-56 and circTRAM1-57 was detected.

Taken together, linear and circular TRAM1 transcripts showed decreased differential gene expression in HR BCa patients and in the higher cancer stage cell culture model. Therefore, TRAM1 RNAs could function as potential tumor markers for a non-invasive detection of BCa in urine of patients. We further note that a robust detection of transcripts in urine is necessary to qualify as marker molecule even for RNA species with a decline in differential gene expression. Based on the qPCR data for TRAM1 circRNAs in bladder cancer cell lines, the expression level of circTRAM1-56 with a high cDNA input of 50 ng per sample is very low. Moreover, the expression level of circTRAM1-57 was tenfold higher compared to circTRAM1-56 but still too low for a stable detection in the limited RNA amounts of human urine. As a potential tumor marker for BCa, TRAM1 transcripts detected with the convergent primer pair in 10 ng cDNA per sample indicated a high expression level in both bladder cancer cell lines and therefore represent a promising marker candidate for detection in urine. For a robust discrimination of healthy individuals and BCa patients, it would be beneficial to find up-regulated as well as down-regulated transcripts to finally calculate marker ratios independent of absolute copy numbers. Therefore, TRAM1 RNA species could be used for calculation of marker quotients with a clear and stable detection of BCa which we recently described^37^.

## Discussion

### Detection of linear and circular TRAM1 transcripts in BCa cell lines

In case of PCR-based analysis of linear and circular transcripts convergent and divergent primer pairs have been designed and applied for transcripts of the translocation associated membrane protein 1 (TRAM1). In case of RT-4 cells and ECV-304 cells the expected amplicon length has been measured. An overview of transcript species of the TRAM1 locus, their abundancies, and their potential topology was obtained by cloning and sequencing of PCR products. We also confirmed the correct sequences of two out of four predicted TRAM1 circular RNAs (circRNA). Even though many different potential circular TRAM1 sequences of various lengths were detected, the correct back splice junctions (BSJ) could only be verified for circTRAM1-56 and circTRAM1-57.

A direct proof of BSJs of circRNAs, i.e. of circTRAM1-56 was provided by BSJ-specific asON and RNase H. In general, evidence of the physical existence of circRNAs is important to exclude artifacts at the level of cDNA synthesis, PCR, or cloning steps. It is conceivable that BSJs of circRNAs could be generated by template switching or back jumps of reverse transcriptase and trans-splicing of RNA molecules during cDNA synthesis^47^. Even though such artificial sequences seems to occur randomly^43^, they account for a significant proportion of detected sequences with non-canonical splice sites^48^. Further, it should be noted that the RNase H-mediated cleavage of circRNAs by specific asON strongly indicates that these asON can be used for the suppression of circular TRAM1 transcripts in cell models and, thus, for functional studies of the biological function of TRAM1 circRNAs.

### Considerations of criteria for evaluation of authentic circRNAs

The experiences summarized in this work led to a specific strategy for specific and separate quantification of both confirmed TRAM1 circRNAs in cells. This included positioning of one primer directly on the BSJ of circTRAM1-56 as this sequence describes the only difference towards linear TRAM1 transcripts and circTRAM1-57. The possibility of a specific detection of circular TRAM1 transcripts enabled validation of circular topology via examination of tandem repeat hypothesis and RNase R hydrolysis.

First, analysis of flanking noncoding sequences provides data on the probability of the existence of TRAM1 circRNA species in cells. For the detected transcripts circTRAM1-56 and circTRAM1-57 we identified invers complementary intron sequences with lengths of up to 465 nucleotides. Hence, base pairing of flanking introns could contribute to the generation of both potential circular TRAM1 transcripts. An extensive base pairing of two introns could significantly enhance the efficiency of the production of circRNAs^49^. Moreover, invers complementary sequences of only 30–40 nts could promote the generation of circular transcripts in cells^23^. As a result, base pairing of flanking noncoding sequences creates spatial proximity of exon ends and therefore facilitates back-splicing of circular transcripts. Secondly, the examination of tandem repeat hypotheses could exclude exon duplications in the genome of cells as reason for false positive detection of seeming BSJs of TRAM1 circRNAs. All divergent primer pairs detecting different TRAM1 transcripts were tested for tandem repeats on the DNA level and showed no signals in the PCR reaction. In general, the influence of tandem repeats in human genome on wrong allocations of seeming BSJs could be rated as statistically not relevant in sequencing data^22^. Furthermore, the hypothesis of unusual exon arrangements as a result of genomic translocations in cancer cells could be predominantly not be confirmed^22^. Lastly, RNase R hydrolysis should verify the circular topology of both potential TRAM1 transcripts. This exoribonuclease leads to an efficient degradation of linear RNA species while circular transcripts are not targeted^44^. Here, quantification of circTRAM1-56 and circTRAM1-57 indicated no reduction of copy numbers after RNase R hydrolysis. This is compatible with the view that the divergent primer pairs used in this study were able to specifically detect circular TRAM1 transcripts. In summary, both transcripts, circTRAM1-56 and circTRAM1-57, respectively, have been verified as authentic RNA species of the TRAM1 gene locus.

### TRAM1 transcripts as potential tumor markers for non-invasive detection of BCa: diagnostic perspectives

Development of non-invasive tumor markers for clinical diagnostics and therapy monitoring of BCa is of great interest since standardized cystoscopy is time-consuming, related to high costs, and may cause health problems^3,4^. A realistic approach for the simple and specific detection of BCa is based on the analysis of the RNA composition of urine. We have previously reported new potential biomarkers based on whole transcriptome analysis of urinary RNA from healthy individuals and HR patients of BCa^37^. One might speculate that linear and circular transcripts of TRAM1 are marker candidates because they show a noticeable correlation with the health status of donors. Thus, future marker development might include TRAM1 sequences.

Further, PCR amplicons of linear and circular TRAM1 transcripts were tested in the BCa cell lines ECV-304 and RT-4 in order to extend the results of transcriptome data of urine RNAs. The linear TRAM1 transcripts showed decreased expression in urine of HR BCa patients and in ECV-304 cells (G3 cancer stage). The high consistency of calculated lg_10_(fold change) values of both differential gene expression analysis and the robust expression level in cell lines suggest linear TRAM1 RNA species as potential candidates for tumor markers with reduced expression in higher cancer stages. Thus, linear TRAM1 transcripts can conceivably be used to calculate ratios of up- and down-regulated marker candidates for BCa patients^37^.

Conversely, the two circular TRAM1 RNA species showed low expression in both BCa cell lines. The strong under-expression of circTRAM1-56 and circTRAM1-57 in urine of HR patients was not reflected in ECV-304 cells. Studies by others mainly showed a general reduction of circRNA expression in tumors^50^ which is suspected to be linked with high proliferation rates and a dilution effect in cancer cells^51^. Further, only 14 up-regulated circRNAs but 42 down-regulated circRNAs in BCa could be identified via next generation sequencing and verified via qPCR^52^. More specifically, for circ-ITCH a lower expression in BCa was detected by others which was correlated with higher proliferation rates and invasion of tumor cells^53^. Hence, the low expression levels of TRAM1 circRNAs in cell lines are compatible with other studies on BCa and different cancer types.

Taken together, linear and circular TRAM1 RNA species exhibited decreased differential gene expression in the higher cancer stage of patients and the G3 stage cell model. To further validate TRAM1 transcripts as potential tumor markers for non-invasive detection of BCa, expression analysis must be investigated in a high number of single urine samples of patients. Additionally, functional studies in BCa cell lines including suppression or overexpression approaches could provide deeper insights into roles and involved mechanisms of linear and circular TRAM1 transcripts in cancer development.

## Methods

### Cell culture

The human urinary BCa cell line ECV-304 was cultivated in Medium 199 (PAA, Pasching, Austria) containing 10% (vol/vol) fetal calf serum (PAA, Pasching, Austria). ECV-304 was originally established from an invasive, G3 BCa of an 82 years old Swedish female patient with a mutant p53 in 1970. It is a defined derivative of T24^41,42^ which we obtained from the DSMZ (Deutsche Sammlung von Mikroorganismen und Zellkulturen, Braunschweig, Germany). The DSMZ is a national repository for microorganisms and cell lines. Cell identity was confirmed by DNA profiling by the DSMZ. RT-4 cells^46^ were cultivated in RPMI 1640 medium (PAA, Pasching, Austria) supplemented with 10% (vol/vol) fetal calf serum. RT-4 cells were used as an in vitro model for differentiated G1 BCa. Both cell lines were cultivated without antibiotics at 37 °C and 5% CO_2_ in a humidified incubator.

For isolation of cellular RNA, cells were washed twice with PBS, harvested and lysed using QIAzol Lysis Reagent (Qiagen, Hilden, Germany) according to the manufacturer’s instructions. DNA hydrolysis was performed using TURBO DNase (2 U/µl, Thermo Fisher Scientific, Waltham, MA, U.S.A.). Total cellular RNA was isolated using phenol–chloroform extraction followed by ethanol precipitation. RNA samples were quantified using a NanoDrop ND-1000 spectrophotometer (Thermo Fisher Scientific, Waltham, MA, U.S.A.).

For isolation of cellular DNA, ECV-304 cells were harvested as described above and the cell pellet was washed twice with PBS. Pellets were resuspended in DNA isolation buffer (100 mM NaCl, 10 mM Tris–HCl (pH 8,0), 25 mM EDTA (pH 8,0), 0,5% (w/v) SDS, 0,1 mg/ml Proteinase K, 20 μg/ml RNase A) and incubated for 12–18 h at 50 °C. The genomic DNA (gDNA) was isolated using phenol–chloroform extraction followed by ethanol precipitation. DNA samples were quantified using a NanoDrop ND-1000 spectrophotometer (Thermo Fisher Scientific, Waltham, MA, U.S.A.).

### cDNA synthesis

Reverse transcription was performed with 2 µg RNA input of bladder cancer cell lines as recently described^37^. RevertAid First Strand cDNA Synthesis Kit (Thermo Fisher Scientific, Waltham, MA, U.S.A.) with random hexamer primer was used according to manufacturer’s instructions. For non-RT control reactions, solutions of RiboLock RNase Inhibitor and reverse transcriptase were replaced by nuclease-free water.

### Conventional PCR

Conventional PCR was performed using DNA Taq polymerase (5 U/µl with Thermopol Buffer, Thermo Fisher Scientific, Waltham, MA, U.S.A.). Concentrations of buffer, dNTPs, primer pairs and Taq polymerase were adjusted according to manufacturer’s instructions. For amplification we used 30–40 PCR cycles and the amount of cDNA template varied from 50 to 250 ng depending on the abundancy of transcripts in cells. Negative controls without template (NTC) and non-RTs were included in the PCR reaction conducted in a thermal cycler (PCR block UNO II, Biometra, Göttingen, Germany). Temperature and time of PCR steps were executed according to manufacturer’s instructions with the exception of the hybridization step which was adjusted to primer pairs. Results of conventional PCR were visualized in agarose or denaturing polyacrylamide gels.

### DNA isolation from agarose gels

PCR bands were excised from agarose gels with a clean scalpel and purified using the QIAquick Gel Extraction Kit (Qiagen, Hilden, Germany). All steps were performed according to manufacturer’s instructions and the final elution of PCR products took place with 50 µl nuclease-free water.

### Cloning of DNA in bacteria cells

For ligation of PCR products into vectors, TA-cloning strategy using the Dual Promoter TA Cloning Kit with pCRII vector (Thermo Fisher Scientific, Waltham, MA, U.S.A.) was performed according to manufacturer’s instructions. Briefly, 1 µl of PCR products of linear transcripts and 3 µl of PCR products of circular transcripts of 25 µl PCR reactions were used for insertion into bacterial vectors. Next, plasmids were transformed into *E. coli* DH5$\mathrm{\alpha }$ cells (Thermo Fisher Scientific, Waltham, MA, U.S.A.) using 50 µl of competent cells and 2 µl of the ligation reaction. Manufacturer’s instructions were followed and the final bacteria culture was spread out on two agar plates containing 30 µg/ml Kanamycin. After overnight incubation at 37 °C, bacterial clones on agar plates were counted and analyzed via colony PCR. Clones with PCR insert were expanded in 3 ml bacteria cultures containing 30 µg/ml Kanamycin. After overnight incubation at 37 °C, plasmid preparation was conducted.

### Plasmid preparation

Plasmid preparation was performed using the GenElute Plasmid Miniprep Kit (Thermo Fisher Scientific, Waltham, MA, U.S.A.) following manufacturer’s instructions. To control quality of plasmid preparation and length of PCR insert, restriction analysis of 1 µg plasmid DNA using EcoRI (10 U/µl, Thermo Fisher Scientific, Waltham, MA, U.S.A.) was performed and visualized in agarose gels together with 500 ng uncut plasmid.

### Sequencing

Sequencing of DNA was prepared using the BigDye Terminator v3.1 Cycle Sequencing Kit (Thermo Fisher Scientific, Waltham, MA, U.S.A.). In case of direct sequencing of PCR products, samples were first purified with ExS-Pure Enzymatic PCR Cleanup Kit (Nimagen, Nijmegen, Netherlands) following manufacturer’s instructions. Sequencing program was performed in a thermal cycler (PCR block UNO II, Biometra, Göttingen, Germany) with recommended temperature and time settings. Subsequently, samples were purified using NucleoSEQ columns (Macherey-Nagel, Düren, Germany) and denatured for 2 min at 95 °C in 1:1 HiDi Formamide (Thermo Fisher Scientific, Waltham, MA, U.S.A.). Finally, sequencing analysis was performed in the ABI PRISM Genetic Analyzer 3130 (Thermo Fisher Scientific, Waltham, MA, U.S.A.).

### Quantitative PCR (qPCR)

qPCR was performed using SYBR Select Master Mix (Thermo Fisher Scientific, Waltham, MA, U.S.A.) in the 384-well plate format as recently described^37^. Primer concentration was 200 nM for a 10 µl total reaction volume with 4 µl template of RT or non-RT sample and 5 µl of SYBR green Master Mix. The amount of cDNA template varied depending on the abundancy of transcripts in cells and is indicated in the table descriptions. The thermal cycler 7900HT (Applied Biosystems, Foster City, CA, U.S.A.) conditions were 50 °C for 120 s, 95 °C for 120 s, and 40 cycles consisting of 95 °C for 15 s, and 60 °C for 60 s. Melting curve analysis was performed. Samples were measured in quadruplicates and negative controls included reactions without template (NTC) and non-RT reactions. Data analysis was performed via the SDS 2.1 software (Applied Biosystems, Foster City, CA, U.S.A.). The RNA level of bladder cancer cell lines was normalized to the levels of endogenous 18S ribosomal RNA which served as an internal control.

### Design of primer pairs

Primer pairs are shown in Supplementary Table S5. Convergent primer pairs for detection of linear sequences of transcripts were designed using NCBI Primer-BLAST (https://www.ncbi.nlm.nih.gov/tools/primer-blast/) with a melting temperature of 57–63 °C (optimum 60 °C, maximal T_m_ difference 3 °C). The PCR product size was adjusted to a range of 70 to 150 nucleotides. Three types of primer pairs were defined for each transcript: Primer pairs in the same exon, primer pairs separated by one intron, and primer pairs with one primer spanning the exon-exon junction sequence. In the last step, primer pairs were checked for specificity of amplification of the target transcripts and problematic secondary structures as well as self-complementarity were excluded. Divergent primer pairs for the detection of circular transcripts were designed according to ‘Circular RNA Interactome’^54^, which was used to identify back-splice junction (BSJ) sequences of a circular RNA. This sequence was inserted into NCBI Primer-BLAST (https://blast.ncbi.nlm.nih.gov/Blast.cgi) and the design of primer pairs was performed as described above. Primer pairs were purchased from the commercial supplier Metabion (Planegg/Steinkirchen, Germany).

### Design of antisense oligonucleotides (asON)

First, linear target transcripts were folded using RNA fold WebServer (http://rna.tbi.univie.ac.at/cgi-bin/RNAWebSuite/RNAfold.cgi) and mfold (http://www.unafold.org/mfold.php) to identify unpaired sequence regions which are not involved in secondary structures. The predicted structures were recorded and conserved unpaired sequence segments of ≥ 10 nts were chosen as local target regions as described by Kretschmer-Kazemi Far et al.^55^. For circular RNAs, only BSJs can be used for specific targeting. Next, a number of five asONs was developed per target region of linear or circular transcript. These asON candidates were checked for specificity to target transcripts and problematic secondary structures as well as self-complementarity. asONs were purchased from Metabion (Planegg/Steinkirchen, Germany) and are listed in Supplementary Table S6.

### Analysis of flanking noncoding sequences of circRNAs

Sequences of introns flanking circRNAs were obtained from Ensembl by analyzing the respective linear reference transcript. Noncoding sequences flanking exons involved in backsplicing were aligned using LALIGN/PLALIGN (https://fasta.bioch.virginia.edu/fasta_www2/fasta_www.cgi?rm=lalign) and the amount and percentage of invers complementary nucleotides were studied.

### Hybridization of asONs to transcripts and RNase H cleavage

For hybridization of nucleic acids, 750 ng of ECV-304 cellular RNA were incubated with 12 ng of asONs and 5× hybridization buffer (75 mM HEPES, 250 mM potassium acetate, 5 mM magnesium acetate tetrahydrate, pH 7,4). Samples were denatured for 3 min at 95 °C and annealing of asONs to cellular RNA took place at 37 °C for 1 h. Next, double-stranded DNA-RNA-hybrids were cleaved by addition of 2 U RNase H (2 U/µl, Thermo Fisher Scientific, Waltham, MA, U.S.A.) and incubation for 20 min at 37 °C. Phenol–chloroform extraction followed by ethanol precipitation was used to purify samples from salts and buffer contents. Subsequently, cDNA synthesis, conventional PCR and visualization of PCR products in agarose or polyacrylamide gels were performed.

### RNase R hydrolysis

2 µg of ECV-304 RNA were incubated with 1 U RNase R (Epicentre, Madison, WI, U.S.A), 40 U RiboLock RNase Inhibitor (40 U/µl, Thermo Fisher Scientific, Waltham, MA, U.S.A.) and 10× RNase R buffer at 37 °C for 30 min. Phenol–chloroform extraction followed by ethanol precipitation was used to purify samples from salts and buffer contents. Subsequently, cDNA synthesis, quantitative PCR and visualization of PCR products in agarose gels were performed.

### Statistical analysis

For statistical analysis of qPCR data, two-sided unpaired *t* test was applied using GraphPad Prism (GraphPad Software, San Diego, CA, U.S.A.). Differential gene expression of BCa cell lines was considered to be statistically significant for *P* values ≤ 0.05.

### Data availability

The datasets generated during and/or analyzed during the current study are available in the NCBI sequence reader archive (SRH) repository, study: PRJNA757762; submission: SUB10259481. https://www.ncbi.nlm.nih.gov/sra/PRJNA757762.

## Supplementary Information

Supplementary Information.
