# Supplementary material for: The human TRAM1 locus expresses circular RNAs
Source: Sci Rep. 2021 Nov 11;11:22114. doi: 10.1038/s41598-021-01548-0 (PMC8586232; doi:10.1038/s41598-021-01548-0)
Supplement: Supplementary file 1 — Supplementary Information. [file 41598_2021_1548_MOESM1_ESM.pdf]

**Title:** **The human TRAM1 locus expresses circular RNAs**

**Authors:** **Josephine Dubois[1,2] and Georg Sczakiel[1,*]**

**Affiliation:** [1] Institut für Molekulare Medizin, Universität zu Lübeck and UKSH, Campus Lübeck, Ratzeburger Allee 160, D-23538 Lübeck, Germany.

[2] current address: Department of Internal Medicine, Division of Hematology and Oncology, University of Michigan, Ann Arbor, MI.

*to whom correspondence should be addressed

**Contact Information:** Tel: +49-451-3101-9050
Fax: +49-451-3101-9054
Email: georg.sczakiel@uni-luebeck.de

**Keywords:** circular RNA, TRAM1, urine RNA, biomarker, transcriptome analysis

## Supplementary Tables

**Supplementary Table 1: Clinical Characteristics of Patients.** This study was approved by the local ethical committee of the Universität zu Lübeck. All bladder cancer patients were classified according to the World Health Organization 2004 grading and risk system. Description: f = female, m = male, C = control group, HR = high risk, HG = high grade, Ta = noninvasive papillary BCa, T2-4 = invasive BCa, G2/G3 = grading of BCa.

| Pool | Patient Number | Sex | Grade | Risk | Stage | Recurrence |
|---|---|---|---|---|---|---|
| C | 48 | f | - | - | - | - |
| | 50 | m | - | - | - | + |
| | 80 | m | - | - | - | + |
| | 138 | m | - | - | - | + |
| | 160 | m | - | - | - | - |
| | 166 | f | - | - | - | + |
| | 170 | m | - | - | - | - |
| | | | | | | |
| HR | 41 | m | G3, HG | HR | Ta | - |
| | 46 | m | G2, HG | HR | Ta | - |
| | 107 | f | G2, HG | HR | Ta | + |
| | 128 | m | G3, HG | HR | Ta | - |
| | 159 | m | G3, HG | HR | T2-4 | - |
| | 164 | m | G3, HG | HR | Ta | + |
| | 183 | m | HG | HR | T2-4 | - |

**Supplementary Table 2: Sequences of spliced transcripts and PCR amplicons for circTRAM1-56 and circTRAM1-57.**

| Transcript | Sequence 5'→ 3' |
|---|---|
| circTRAM1-56 | **Spliced transcript: (383 b)**<br>AAGAACAAGCTACTGAATCAGTGTCCCTTTATTACTATGGCATCAAAGATTT<br>GGCTACTGTTTTCTTCTACATGCTAGTGGCGATAATTATTCATGCCGTAATT<br>CAAGAGTATATGTTGGATAAAATTAACAGGCGAATGCACTTCTCCAAAACA<br>AAACACAGCAAGTTTAATGAATCTGGTCAGCTTAGTGCGTTCTACCTTTTTG<br>CCTGTGTTTGGGGCACATTCATTCTCATCTCTGAAAACTACATCTCAGACC<br>CAACTATCTTATGGAGGGCTTATCCCCATAACCTGATGACATTTCAAATGAA<br>GTTTTTCTACATATCACAGCTGGCTTACTGGCTTCATGCTTTTCCTGAACTC<br>TACTTCCAGAAAACCAAAAAA<br><br>**PCR amplicon: (83 bp)**<br>CTACTTCCAGAAAACCAAAAAAAAGAACAAGCTACTGAATCAGTGTCCCTT<br>TATTACTATGGCATCAAAGATTTGGCTACTGT |
| circTRAM1-57 | **Spliced transcript: (447 b)**<br>ATAACGGCAAAAGCTTCTATCATTTTTGTTACTCTTCAGTACAATGTCACCC<br>TCCCAGCAACAGAAGAACAAGCTACTGAATCAGTGTCCCTTTATTACTATG<br>GCATCAAAGATTTGGCTACTGTTTTCTTCTACATGCTAGTGGCGATAATTAT<br>TCATGCCGTAATTCAAGAGTATATGTTGGATAAAATTAACAGGCGAATGCA<br>CTTCTCCAAAACAAAACACAGCAAGTTTAATGAATCTGGTCAGCTTAGTGC<br>GTTCTACCTTTTTGCCTGTGTTTGGGGCACATTCATTCTCATCTCTGAAAAC<br>TACATCTCAGACCCAACTATCTTATGGAGGGCTTATCCCCATAACCTGATG<br>ACATTTCAAATGAAGTTTTTCTACATATCACAGCTGGCTTACTGGCTTCATG<br>CTTTTCCTGAACTCTACTTCCAGAAAACCAAAAAA<br><br>**PCR amplicon: (118 bp)**<br>CAGCTGGCTTACTGGCTTCATGCTTTTCCTGAACTCTACTTCCAGAAAACC<br>AAAAAAATAACGGCAAAAGCTTCTATCATTTTTGTTACTCTTCAGTACAATG<br>TCACCCTCCCAGCAA |

**Supplementary Table 3: Analysis of intron sequences of circTRAM1-56.** Noncoding sequences were obtained from Ensembl by analyzing the linear reference transcript TRAM1-201. Flanking introns of circTRAM1-56 were examined concerning inverse complementary sequences. Local alignments were performed using LALIGN/PLALIGN and the three best sequence matches are shown with quantities and percentages of complementary nucleotides (nts). Positions of sequence matches of intron sequences are highlighted in bold letters.

| **Intron 2, Sequence 5'→ 3'** |
|---|
| GTAGGTAATTTACGCTAAGAATTTTCTACGTAAATAGAAGTTCAGTTGAACAAACTTGGAAATTTTCATGAAT<br>GCTAGGTCCTGCGTTAATTAATCGTCAGAGATCTCATCCATGATGTGACTGAATGTTATTGTGATGATTTTAT<br>AATACAAGATCTGATGTTTTTGATTGACTTTACCCTGTATATGTTTTCTGTTTACTATATATTTTTGTGAATG<br>GACTTGAAATTCCATAAAATAATTTTGTTTTGCTTTCTCTGCTTTCTGATTTTTTCCTAAGAAAATTATAAGA<br>AAAGATCTTTTTAAAATTGTTTAAATGTCATTGTTCATTCAAGACTTCTAAAAGCACAATGGCAGATTTAGTT<br>TTTCTGTAAGTTACATTTTGGACCTGTAATAGCAATCAATCCTTCTAAGATTATTTTCACTTTTTCCTGTGCT<br>CTACTTTTAACAATTATTTTGATTCCAGAAAATGTGTTAGTAAGCTCTAGGAATTATCTGGTAATAGCTCCAA<br>ACTATTAGGTTTTATTTCAAAAATCTTATTTCTTAATCTATGTAAAAATTAGGTGTTTCTTGGAAGTTGATTG<br>TAATTTAATACAGTGATATAATTAACTAGCATGGGTGAGGGATGGGGTATTTTTTGGTTGAGGCAGTAAACCT<br>GCACAGTTTTTTATTTAGAATGTAAGATAATATAGTATACCACATTTAGTTGGGGTTTTTTGTTGTTGTTGC**T**<br>**GTTATTTATATGTGTGTTTTTAAGAGATGACAGGGTCTTGCCAAATGTGTTACCTAGGCAGGAGTGCTGTGGC**<br>**GTGATCGTAGCTCACTGCAGCCTCCAGCTTCTGGGCTCAAATGATCCTCCCAGGTCAGCCTCCCAAGTAGGTG**<br>**GGACTACAAGGGCACACCACCACGCCCAGCTAATTTTTCAATTTACTATAGAGATGGAGTCTCACTATACTAC**<br>**CTAGGTCAGTCTTGAATTTGGGCCTCAAGCAATCCTTGCCTCCCTAAGTGTTGGGATTACAGGGGTGAGCCAC**<br>**TGCATCTAGCCCCTGAGTTGGGTTTTTGTTCAGTTATGTACAGTTTTGTAAATATTTAATTTAGTGATTTAGT**<br>**T**ATCAGGTTACTCAAAACTACAATACTGTTTTGAAATTTGAATATGCCAGAGAAGAATAAAGCTCTTTTATGT<br>ATATGTTCCCCATAAACATAAAGTTTGAGAATAAGTGTCAGCAATTCACAAGTATTTGATAAAGATTTGTTTT<br>GAAAAATTATAGCAATTGCAAGGATTAAAAACAACTTTAGTAGTTGCCTTTATCTCTGCTGCTTATAGATCAA<br>AACTAAATCAAATACAGGTCAAATCAGCTTTTTCATCAGAATCATAATTTAGTGTGTAGTTAGAAAGTAGAAG<br>TAGAAAGTTTCAAAAGCCCTTTGAAAAAAATAGGTCAGTAGGCTAAGTATAACTTTATCAACCTAATTTATAC<br>CCCTGGGATAATATCAGTTTATTGAGTGTGGCCTCCTCTTCCCTGTTTTTTGGTCTTTGGTGGCTGTGACTAA<br>CATGTGTCAAGTCTTTGTAAGTAATTTCTAAAGCTTTAGATTTCTAGATAGTTTTGATTTTCATTGCCCCTCT<br>AAGACTCAAGATGTCCAAGACCACGTTATTTATGATGTTAATATAGAACTTTTTTATTAGGTTATTCTTCTTT<br>GTTTCCATAACTATGTTGTACTTAATGCTGTTTTTATAACCTTGTGTATATTTTTGTGTATTTGTGTCCTTTT<br>TGGTCTTCAG |
| **Intron 6, Sequence 5'→ 3'** |
| GTAAGCTGGGATTTTGTAGTGTTAAAAAATGTCTTGTCATTTTTTTTTTTGAAAAATTTTTCTAAAATTAAGT<br>ATTTTCTTACATGTTTGCCCTTCCTCCATTCTTCTTACCTCAACTCTTACCCCATCATTCTCCCACCAACCCT<br>CTTTCCACAAAGCCTAAAATCCTTAAGCATTTCTTGAAAAAAAAAAAAAAAAAAACCTTCAGTCAGTATTTTAG<br>CTTAGATTTTTTACATTTGATATGTTAATGGCTTGTCTTAAAATTATGTTAGTGGTAATGTAAATTAAAATTT<br>AAACTAATTTTTAAATGTAATATTTGTTTTATCTTACTGATATTCTTAGCAAATTATTTCACAAGTGAGCCAA<br>AAAGGATTAGTCACATTACAACTAACAGTTCTGTGTGTAAGTCCTAAAGCAAAATTCAGTATTGGAAAAAAAA<br>GTAAAATATATTTAAATATTCACCAGCAAAGCCTCTCTTGAAATGGGTAGAGCATTCCCTTTTTAGCATGTGG<br>AAAGGATATTTGTTTGTTTTATTTGAACAAATAAGTAAACTGCTGGAGCACTGCAAAATCCTCCTTCCTAGTT<br>TTACCCTCAGTGCTTCTGAAGTCGGGTTCCATGCTATCACAAAAGTCGTTTTCTTTACCCTCTCCCTTCTGAA<br>ACCTGTATTGGCTCCCTATTACTTTAAACTAGAGTAATGCTGCTCAACTGTGCCCTGAGAGTTTCTCCCATCG<br>CCACCATCTTTTCTCGGGGACCTCTTTCTCTGCTTACTCCTTATGCTTCATGCACACTGATAGTCTCTAGTTT<br>TGTAATCCCAGCACATCACTTCTTTTTCCTGGAACACCTCTTCCCTCGGATAAATTAATCAAGTACTTTCATT<br>TCCCTTTTTTCTGGGAGTTTTTTTTTTTTTTTTAATTCTCATATATGAAATCCTATGTGGGATTTTACTTCAA<br>AATAATACCAGGTGAAGGAAAGTGAAGAGGGATATAGATGAAACAAGTTTGATCGTTTATTAATAATGATTGA<br>GTTACGGGTATGTCAGAATCTTTTCTGTCTGTTGTGTATATGTTTACATTTTTCCGCATTTAAAATTTCTTTT<br>TTTTTTTTTTAATCCCCTTTGAGAAACCTGGAGTGAAAGTGTGACAGCCAGTCTGGTGACTAGTTTATCTTTG<br>CCTACTCAAAAGCCATAAAACACTATTTTTATGTGTGCTACAAATAGTTAATTCATGGACTAGGCACTTGATT<br>TCCTACTGTTTCGTGTCTGTGTTCTGTGTATCTGTCGTTACTCATGTTGTCTGCTAGGATTAGAAAACAGCAC<br>CAAATTAAAGGCCATATGCTATGGTACTTAAGTTCTTTTTATGCTGATTTCTTTTATAAAGTAGATTGTTTCT<br>CCAGCTATATTGATAAGGAATAGGATGAAGACCTTTTATTTTTAAACCCTTGATTTAGAACTGTCTTCAGATA<br>AAATTGTTAGGCCCAGATTCCAGTAGCCCTTCCATGTTTAGTAAGCTCCTAATGTGAATCATAGATATTGTGG<br>AGCTGTACTGTCCAAAATGCTAACTACTAGTCACATCTACATGAACTTAAAAAACAAAAACGAAATTGACTTT<br>TTTAGCCCCAGTAGCCACATTTCAAGTGCTTATTAGCTACTGTGGCTAGTATGTACCATTTTGAACAGAGGAG<br>ATATGGAACATTTCCATGATTGCAGAAAGTTCTATTGGACAGCGCTACTGAGAGTATCATAGTTTCTTGATGT<br>CTTAGTAGCATGGCTCAGATATTTAAAGCCTTCTTAAGGTATTTGTATACTCATGAACAAATACCTCTGGATT<br>TCATTATTAATCCATGTCTATATCTTCTTCATTGACTATGGTAAGTAAGAAGGGACATTTCAAAGTAATTGTT<br>CATTTTAGTTCCTGAAGCTCTTTTCTTATCAGTTCCTGCCAGATGGCACTTCTCCAGAAATGCAGTCTAATTT |

TTATGGAATAATTTGCATAACTCAGAACTTAACTGAAACTATTTTTAACACCTGCATATGACAGTTGAAAGTA
ACTGTTTCAAGTAATTAACATAATTTGAAAGTAGCAGCTGCATATGTGTTAAACAGTTTCTTAAAATTTACTC
TATGAAAATTATTTTTGGAACTCTTTACTGGAAGAAACTAGTACAGTGATTGTTTTGAACCAATCATACAGAC
AAATAGGAAAGAAAGGAACGCTGTTAGTAGCAGTTCAAGAGGTGTGTTAGTCACAAATAACTTAGTTTCTTTC
AAAGATAAAACTTAAATTACATATACTGTGTATATAAACAGTTCTCCATTTTGCCCATGAGAAGATTCAAAAT
ATTTCGTAGACAGCTTGAATATATGAGCTTTTAGGGAAGTCATGAAATAGCCAAATGTTTTATTTCTAATATT
CAATTTTAGTTTAATTATACATACATATAATTATACATACCTATAATTGCATATCATATAAAGTATTAGAAAT
AGTATTTTCCCCCTTCCTACTTCATGAAAGTTTCATTACACTTTTCATATGCAACTTTGATGTTAGTATCTGT
CATTCATCATCACTAGAACCACTTTTTTTTAATCTGATGACATTTTTTCCACATCATGACGTCATCTTCTGTC
TCACTGGTTCCTTCATTAGACCTTATAAACCTTTAAAAATGACATTGATTGAAGTTTTCGGGTGTATGTGTCT
TACGCAAACATTTATTTGTCAAGATAATTAATACAGAGCTCTATAATGCAGAGCTTTGTAAAGATTTAATTAT
AAGATGTACCATCTGGGGTTTGGGGGGAGATCTTTGCCTTATATTTGGGGCATATATTTGATATTCAGCTTTG
TTTATTTTTCAGTATAGAGTAGTCAAGAAATGTGTTTTTTATAAAAATAAATTTTTTGAAGTATGTTATATTT
TTTAATAAACAGATGTTGGAATAACAACTGAAAATGGCACTTGAAGTTATTTACGATTTTATAATTTGTTATG
ATTTCACCCAGCCTTCTCTGTTCTCTCCCTTCCCCCACAACCCATGTAAATGTGTACGTCCAGAGACCATTGT
GCTGGTGCTAGGCAAGGCTTTGTTGTCGGCCTCAGACATGTGAACACCGTGCCATCGGGGCTGTAACCCCAGA
GGAAATGGAGAACATTGAGGGAGGCTGGCCTACCACAGGAATACTAAAAAGTAGCCATTTTCTAGATAAAATT
TATCATTTTAAAAATCATGGTAAGCCAGGCATGGTGGCACACACCTGTAGTCCTGGCTACTGGGGAGGCTGAG
GCAAGGGGATGGCTTGATTCCAGGAGTTTGAGACCAGCCTGGGCAATGTAATGAGACTCATCTCTAAAAATAA
AATAAAACTCATGGTACACTACCCCTACTTCCATGTATTTTTTGTAATCATTTGGGAATACCTTTATGTAGGG
TGTTTGTTAAGTTAGCCACAATTCCTACCACCCCTTGTGGTATTCTACTCATCTCTTTTTTTTTTTTTATTATT
ATTGTTGTTGTTATCCCCATTATACAGAGAGATTAATTAGCTTGCCTAAGGTCCCTCAGCTAGTAAGTGACAC
AAGTATTTACCCATTCTCTTACTAATTTACTTTTTCTGTGGTAAGCTTTGAGTGTACAAACACTGCTTTAGAG
ACACTAGTCAATTTGTGCAAAGCTGAATGGACCATTACTTAGAAAAAGGTTTCTTGATAATCCTTTTTGAAAG
CAATAGATACTATTTTTAAAACTTTTGCATAGGCATTTGATGGTTGTGAAGATAGAACCTAGTGATGGCATTT
TACCCTCTATTATTATTGACTCGGTTGACTATCCAAAAGGAAAGGCTACTGGGCACAGTGGTGCATGCCTGTA
GTCCCAGTTACTCAGGAGGCTGAGATAGCAGGATCACTTGAGCCCTGGAGTTGGAGGCTACAGTGCAGCATAA
TTGCACCTGTGAATAACCACTGCACTCCAGCCTGAGTAACATAGTGAGACCATGTTTCTATTTTTTAAAAAGG
AGGTATGGGGCTAAGATACAAAGTTGAATCTGAACTTAGGAAAGAAACTCTATGGATTTTTCCTTAGCTAATG
TCAAGCACAACTGCGCTCCACCTAATTGGGTTGAAATTTAAAGACAAAAGATCAGAAATGGAATGGCTTTTTG
AATTATAGTTTGTTTGTAATTTATAATAGTGTTTTTAGTCTTGTCAGAGAAGAAAAGAGTAGGCAGCTTAGGT
TCTCTGCTAATTGACCAGTGTGAATTTTAAAAACTTACTTGTATTTCTTTTAAATTAAAAAAAAAAAAAATCCT
CCTAAGTAGTTACATTACATTATAGTTTTTCCAGCAAATTAGTTTTTTTTTTTTTTTACATTGCCTTACCCAA
GTTTTTATGCAATGAAATGAGTTCAATCAGCAGTTAAAAACTAAAACGCTTCCCTCATAAGTTTTAAGGGTTT
TCTTGGTCTGTTTTCTCCTCTGAAATGTGGAGCATTGAGTAACACATATGAAAGAATATGGCAATATTTTTTA
TACCTAGTCATTTATGTGCCACCTTCCTGCTTTACCATATTCACATACTACTAA**TTCATACTTTTGACATTTT**
**AATTATTAAGGTATATTAAATAACAAAATATTTTGGGTTTTTGTTTTGTTTTGTTTTGTTTTGAGACAAGGTC**
**TTGCTCTATCACCCATGCTGGAGTGCAGTGGCCACAATCACGGCTCAGTGCAGCTGCAGCCTCAACCTGCCAG**
**GCTTAAGCAATCCTACTACTTCAGCCTCCCAAGTGGCTGGAAGTACAGGCATGCACCACTATGCTTGGCTAAT**
**TTTTTGTATTTTTTTAGAGATGGGGTTTCGCCATGTTGCCAAGGCTGGTTTCGAACTCCTGGGCTCAAGCAAT**
**CCACCTGCATCAGCCTCCCAAAGGGCTGTTATTACAGGCGTGATCCACCACTTTATTTAAAATTTTAGTTACA**
**GTTAAATAAAAGTATTTCATTGTGTACTGTCTGCAATCATTTTGTT**CAGTACCAATGTACACACCACACTGGG
AAACACTGGCATATATATAGTGGCACTTCTACATGCATTGTACATCAAATTGCATTGATAATACTAAGTGAGC
TTTTCTGAGATGATTATAACGGGAGAAGAGAGGTTCCCTTGTGGTTGCATGTTATCTCAACACCCTTTCTCCT
TCCTTTTGTAAGCTGAAATATTTCATTGGAAATTTACAAATTATGTAGAGAGGATCTTAAGACCGTCTATTTT
CAGAGATACTTCTCTCTTCCATTGGAAGAATGACTGTAAACTTGGTATCAATTTTCCTAAGAGTCCCAAAATA
CAGTACTGTAAATTGTAATATTCAGACTGTCTTATGTTCTACCTAAAACTTGATCTTTGCGCCATGCTTACTA
GAGAAGAGTTTTCCATTTCAAAACTAGACTTCAAAAAATCTCTACTCCTCAAAAGATTTGGTGAAGTAGCTCT
CAGTACTGAAACACAGAGAAATGGAGGACCAGTAGACCTGCACTCAACTGTGCTAAATGCAAGAGATAAGTTT
AGTGAGGCTGCAGAGGAACACAGTTCAGCCTGCCAGCAGCAACCCACCATTTCCATCGTTCTAATTAGTAGCT
GTAGAACATGAATAGTACTAACTAGACTAATATAATCCTTGTAAATATACATACGAAGAAAACTGCTACAACA
AAGGAAATTAGAAATGAGGTATAGGTGTTTCATTAGTCAGTAGGCTGGCCTGTTGAGCTTATCACTGTCAGTG
TTTTGTAAACATTTTTACTGCAACCAAAGATGAAAAACTTTTTACATCATAATTCATTGTGTGTTTGTGTGTG
TGTAAAATGTGGAATACAATTTTCATAATACAATGTGCTATATACTCTAGCA**TTTTCTTTTTCTTTTTTTTTC**
**TTTTTGAGACAGCGTCTGGCTCTGCCGCCCAGGCTGGAGTGCAGTCACAATCCTAGCTCACTGCATCCTTGAA**
**CTCCTGGGCTCAAGCAATCTTTCTGCCTCAGCTTCCCAAATAGCTAGGACTACAGGCACATGCCACCATGCCT**
**GGCTAATTTTTAAAACATTTTTTTGTAGAGATGGGGTCTCTCTCTGTTGCCCAGGCTAGACTCAAACGCCTGG**
**CTTCAAGCAATCCTCCTGTCTCAGCCTCCCAAAGTGCTGGGATGACAAGTGTGAGTCATGGTGCCTGGCCACA**
**TTTTCTATTTTATTTCATGTTTTAAAAGAT**GCTGGTGTGACCCCTAAATTGATTTCATGA**TCCACTAGTAGGT**
**CTTTTTTTTAAAAGACAGAGTCTTGCTCTGTCACCCAAGCTGGAGTACAGTGGCATGATCATAGCTTACTGTA**
**ACCTCGAACTCTTGTGTTCAAGTGATGCTCCTCCTGTGTCTGCCTCCTGAGTAGCTGGGACTACAGTTGCATA**
**CCACCACGCCTAGCTAATTGTAGTTTTTGTAGAGACAGGGTCTTGCTGTGTTGCCCAGGTTGATCTCGAACTC**

**CTGGCCTCAAATGATCCTCCCACCTCGGCCTCCCAGAGTGCTGGGATTATAGGCATGAACCATCACACCTGGC**
**CCCACTAGTGGGTCTTGACTTGTGTTTTCTAAA**AATACTGTTTCTGAGATAACCAGCTCAACTAGATAACTGT
GGGAGAAAAAAAGAAGTAAAAATAAAATCTAGGTACTTGGGCTTGCAAAGTAATTGAGGAAAAAGAATATACA
ATTAAGGAATAGTATAAATGAGAATACAAAATGTTAAACTTGTTGTAATGACTGACAAGGGACTAAAGCATGA
GCTGAAAATGTGAATCCTATTTTAGGTAAACAGAAGTGTCTCTACTACTATGAACATAACAATTTTAATATTA
GAGGAACATCAAATTGTCAGCATGTAACTATGATTAATATTGTGTCATTTAATACACATTAGAAAAAAGAGCC
ATAGATCTTAGCTCTAAAAGGGATTTTGAGTTTATCTGATTGAAGCAAATCTAAGTATTCACATTTTATGGAT
GAAATAACTGAAGCCAGCAGAGGTTAAGTGACTTGCTAATGCTATATACCTAATTGAGGATGAGAGATTGCGG
TGGGGTGGGAGCAGAATAGCTTTCAGATCTCCTACTTCTACATTTTTCACTTACTCTTAAAGAACAGAAATGA
GCCAAGCACAGTGGGTCACGCCTGAAATCCTAACCCTTTGGAAGGCCAAGGTTGGGGGTTCTCTTGAGTCCAA
GGTTTTGAGACCAACCTGGGCAACATAGCAAGACCACACCTCTTAAAAAAAGGGAAAGGAAATGTTTTAGCAT
GTTTTAATCTATAATGTATTTTTTTACAG

**Inverse complementary sequences, sequence match 1**

67.4% identity (67.4% similar) in 334 nt overlap (6277-6603:745-1065)

Intron 2:
GTGTTTTTAAGAGATGACAGGGTCTTGCCAAATGTGTTACCTAGGCAGGAGTGCTGTGGCGTGATCGTAGCTC
ACTGCAGCCTCCAGCTTCTGGGCTCAAATGATCCTCCCAGGTCAGCCTCCCAAGTAGGTGGGACTACAAGGGC
ACACCACCACGCCCAGCTAATTTTTCAATTTACTATAGAGATGGAGTCTCACTATACTACCTAGGTCAGTCTT
GAATTTGGGCCTCAAGCAATCCTTGCCTCCCTAAGTGTTGGGATTACAGGGGTGAGCCACTGCATCTAGCCCC
TGAGTTGGGTTTTTGTTCAGTTATGTACA

Intron 6:
TCCACTAGTAGGTCTTTTTTTTAAAAGACAGAGTCTTGCTCTGTCACCCAAGCTGGAGTACAGTGGCATGATC
ATAGCTTACTGTAACCTCGAACTCTTGTGTTCAAGTGATGCTCCTCCTGTGTCTGCCTCCTGAGTAGCTGGGA
CTACAGTTGCATACCACCACGCCTAGCTAATTGTAGTTTTTGTAGAGACAGGGTCTTGCTGTGTTGCCCAGGT
TGATCTCGAACTCCTGGCCTCAAATGATCCTCCCACCTCGGCCTCCCAGAGTGCTGGGATTATAGGCATGAAC
CATCACACCTGGCCCCACTAGTGGGTCTTGACTTGTGTTTTCTAAA

**Inverse complementary sequences, sequence match 2**

65.2% identity (65.2% similar) in 351 nt overlap (5893-6235:731-1068)

Intron 2:
TGTTATTTATATGTGTGTTTTTAAGAGATGACAGGGTCTTGCCAAATGTGTTACCTAGGCAGGAGTGCTGTGG
CGTGATCGTAGCTCACTGCAGCCTCCAGCTTCTGGGCTCAAATGATCCTCCCAGGTCAGCCTCCCAAGTAGGT
GGGACTACAAGGGCACACCACCACGCCCAGCTAATTTTTCAATTTACTATAGAGATGGAGTCTCACTATACTA
CCTAGGTCAGTCTTGAATTTGGGCCTCAAGCAATCCTTGCCTCCCTAAGTGTTGGGATTACAGGGGTGAGCCA
CTGCATCTAGCCCCTGAGTTGGGTTTTTGTTCAGTTATGTACAGTT

Intron 6:
TTTTCTTTTTCTTTTTTTTTCTTTTTGAGACAGCGTCTGGCTCTGCCGCCCAGGCTGGAGTGCAGTCACAATC
CTAGCTCACTGCATCCTTGAACTCCTGGGCTCAAGCAATCTTTCTGCCTCAGCTTCCCAAATAGCTAGGACTA
CAGGCACATGCCACCATGCCTGGCTAATTTTTAAAACATTTTTTTGTAGAGATGGGGTCTCTCTCTGTTGCCC
AGGCTAGACTCAAACGCCTGGCTTCAAGCAATCCTCCTGTCTCAGCCTCCCAAAGTGCTGGGATGACAAGTGT
GAGTCATGGTGCCTGGCCACATTTTCTATTTTATTTCATGTTTTAAAAGAT

**Inverse complementary sequences, sequence match 3**

61.9% identity (61.9% similar) in 465 nt overlap (4581-5010:658-1097)

Intron 2:
TGCACAGTTTTTTATTTAGAATGTAAGATAATATAGTATACCACATTTAGTTGGGGTTTTTTGTTGTTGTTGC
TGTTATTTATATGTGTGTTTTTAAGAGATGACAGGGTCTTGCCAAATGTGTTACCTAGGCAGGAGTGCTGTGG
CGTGATCGTAGCTCACTGCAGCCTCCAGCTTCTGGGCTCAAATGATCCTCCCAGGTCAGCCTCCCAAGTAGGT
GGGACTACAAGGGCACACCACCACGCCCAGCTAATTTTTCAATTTACTATAGAGATGGAGTCTCACTATACTA
CCTAGGTCAGTCTTGAATTTGGGCCTCAAGCAATCCTTGCCTCCCTAAGTGTTGGGATTACAGGGGTGAGCCA
CTGCATCTAGCCCCTGAGTTGGGTTTTTGTTCAGTTATGTACAGTTTTGTAAATATTTAATTTAGTGATTTAG
TT

Intron 6:
TTCATACTTTTGACATTTTAATTATTAAGGTATATTAAATAACAAAATATTTTGGGTTTTTGTTTTGTTTTGT
TTTGTTTTGAGACAAGGTCTTGCTCTATCACCCATGCTGGAGTGCAGTGGCCACAATCACGGCTCAGTGCAGC

TGCAGCCTCAACCTGCCAGGCTTAAGCAATCCTACTACTTCAGCCTCCCAAGTGGCTGGAAGTACAGGCATGC
ACCACTATGCTTGGCTAATTTTTTTGTATTTTTTTTAGAGATGGGGTTTCGCCATGTTGCCAAGGCTGGTTTCGA
ACTCCTGGGCTCAAGCAATCCACCTGCATCAGCCTCCCAAAGGGCTGTTATTACAGGCGTGATCCACCACTTT
ATTTAAAATTTTAGTTACAGTTAAATAAAAGTATTTCATTGTGTACTGTCTGCAATCATTTTGTT

**Supplementary Table 4: Analysis of intron sequences of circTRAM1-57.** Noncoding sequences were obtained from Ensembl by analyzing the linear reference transcript TRAM1-201. Flanking introns of circTRAM1-57 were examined concerning inverse complementary sequences. Local alignments were performed using LALIGN/PLALIGN and the three best sequence matches are shown with quantities and percentages of complementary nucleotides (nts). Positions of sequence matches of intron sequences are highlighted in bold letters.

| **Intron 1, Sequence 5'→ 3'** |
|---|
| GTGAGCCCGCCCCGAGTCCCAACCGCCTGCCCCGCCACCTCCAGCCCGGGCCCGGGCTCCAGATGCCAGCCCC |
| GGCTTCTCCGCCCAGGACGCGGGGGGCTCGGGCCCGGGGTGGGTGCAGACCCCCAGTCCCTGAGGTGCAGGCC |
| TTCCCTGCCCTGCCCCACGTCGGCGGCAGCTCCCCGGCGGCCGGCCCGGAGATGCAAAGTCCCGGTGGGCCTC |
| ATCGCGGCCCGCAGGGGGTGGGGACCGCCGCGTCGCCCCCTTCCTGACCCGCCGCCGCCCCCTCCCGCAGGCT |
| GCGCGGCGCAGTTCCTGGTTCCCGCGAAGGGTTACGTGGCAGCCGGCGAGGGGCCTGCGGGCGGGGGCGGCCC |
| CTGCGCTGCCGAGCTGGCTCGGGGCGGGTGGGCGGACTTGGGGGGACTGCCAGCCTCGGCCGCGGTGAGCGTC |
| AGCCCCGCGAGGGCCGGGGAAGGCCGAGGCCCGGCCCCCGGGTCTGAGGAGTGCCGGCCTGGAGACCACAGGC |
| CCCGAGTGACAGGGGAGCCCCCGGTAGCTCCGAGATTGGGGTGAGCGAGAAACCGGAGTCAGGCATCTGCTTT |
| CGACTTGAAGGGCGGTGGCCGCCGGGCACCGGCGCAGTGGAGGCGCGCCAGATTGTTTTCATTTTTGTCTTTT |
| AAACTGATATTATTACCAGTTAACGAACTTTTTGCCCTGACGCTTTAAAGAAACAAGGTCTGATGGATAAATC |
| GAGGTTCTGAACAGCGGTGCCAAGGAATCGAGTAATTTTGTTTCTTGAAGTAGGAGAGGATGGGAATGTCAGG |
| AGTGTGTAAATTCTCTTCCCACGTATCTAGTATTTTGATGACTTATTTAACAGCTGTTGAATGTGGATGATAA |
| ACTTTGCAAGGTGAACGAGTAACATCCTACGTTTTTCCCCTCGCTCCCCAATACACACGCTCATTCCTTTGCT |
| TTTTTCCCTTTTTAATTTTTCTCTGGAGCCTTTATGTCTCAGTAAATATACAAATTATTTTGCTCCTGCCTTG |
| TTTTGTCTCCTCTCTCCTGCATGTAACAAGTGAGAGTAGAGATTTTGGAGTATTTTTACGAGTCTGTATTTCC |
| AGAGTCTAGGATAGTGCCTGGGACATAGGAGTGCTTAAAAAAAGATGAGCGACGTTAAGAATCCTTGAGAAAA |
| CCTAATGGAATGATATTACCAGTGACCAAAAGGTGAATACATTTTCTTTTTAATGGAGCATATTTGTTTTTTT |
| TTTTAATTTTAAAATTAAATAATATTTTAACCTTATTTTCGCCTCTCATCAAATGGGCTTTTCTGGATCTAGA |
| TACAGATATTATTTACTCCTGATTGTGTCATGCATGTTTGAAACCACTGCTAAGGAGGTAGCAGGTATAGTTA |
| AAGGGTAGGTAGAAGGAAACTAACATTCTCAAGCTTTAAGTCTGGGCTGTGCCACCTACCTACTGGGTGACCC |
| AAGTCTGAGCCTCAATTTTTCTGCTCTTTATCAGGAAATAATATCACCTGTACTGTTCTCCTCACAGCCATTT |
| TTTGCAAGTATCAGATGATATGGTGTGAAGGTAAATACAGTGCTAGGCCTGGTGCTCTGGCTTATGCCTGTAA |
| ACATCCCAACACTTTGGGAGGCCAAGGCAGGAGAATCACTGGAGCCCAGGAGTTCGAGACCAGCCTAGGCAAC |
| ATAGGGAGACCCTGTCTCTACAAATGATTTTTTAAAAATTAGCCAGGAGTGATAGATAGCACGCACCTGTGGT |
| CTCAGCTGGGCGGCTGAGGTGGGATAATTACTTGAGCCCAGGAGGTGGAGGCTGCGGTGAGCCAAGGTTTCAC |
| CACTGCACTCCAGCCTGAAGGACAGAGCAAGAAGACCCTGTCTCAAAAAAAAACCCAATGTTAAACATTTGTAT |
| TCACTTTCTGAATTTGATACTTGCATGTACTTTCTGCTTTGACCTTAAAGTAGTACTTTAATATATGTAGTAT |
| ATTCAATTTTCTGTAACTTGTCATAAACCATCTCCACATGGTAAGAATTCCTGACTTTAAAAACATATTTTAG |
| GGGTACTGTGACCTGCAAGGTATATTTGCAGTGCTTTGAATGAAATGAAATATAAATCAGAAATTCTGACACA |
| TAGTATAACTTTGATTTTATATAAACTATTTTACATTCTCTTACTATATTTCTAGGGAAGTCCACCACCTATA |
| CCTTGCCTCACATGTTAGTGATTTTTTTAAACAGTGGATTTTCACCTTTATATAAATTTATAAAATTTGAAAA |
| TCTAAATAGACAAAAATACTAAATAGAGGTTTTTAAGTCAATTCAAAAGTGAGTGTTAAAGTGATACACAAAA |
| GTGAAGTTTGTGCGAGTTCTTCCTTTTTCTTACCATATATTAAGGTGTTAGGATTTCAAAGCATGTTATCTAT |
| ATATAGGACTTTGTAGACTAGGTTAGTTTTGCTATTCGACTTTTAAAACAATTGCTGCTGAATCTCAAAGTTA |
| ACATAATTATAGAATTACAGTACTTGTGCTAGAATAATAGAGAAATTGATTTTTTTTTGCAGAACTGTTAAGC |
| CTGTTGTGCAGATACATTGAGGAAATGTCAGAGGATTTATGGTGACATTGACAAAGAAATTCAGAAAGAAAGA |
| AATACTGTATTACTAGCCAAAATGAAGAAGCAATGGAGATACACTGTAAAAGCATGGATTCTGGAGCTGAAGG |
| GATTCATGTTCAAGTTAGGGCTCTGCAGCTTGCTAACTGGGTGACCTAGGGCAGCTCAGAACTGAAGTTTCCT |
| CATCTGTAAAATGGGGCTTATTGGGTTACTCTGACAAATTAACCAATTTGTATTATGCCTAGTTCAATACTTG |
| ACTTCCAAAAGGTGTGCAATAAGTGGTAGCTATTATTATTCTTTAAAAACTCACCAAAATTGGGAAATAGAAA |
| GCATTTTTATGTGAGAGAAGGGCCTGGAATCTCTTTAGTTAGCATCTAAAGAGATTATACATACTGAAAAGGC |
| TAACAAAGTAGTCTGTTTGGCAGTAATGTAAGAGTTTAATTTCCAGGGAACTTCACATTAATCAAAAATTGCT |
| TGTAAATTTTGGAATCATAGTTTTATTACAGTAAATATCTGTCCACATAATTCAAATCATAGTATACAATGAA |
| TGGCTTCATATTTGAGTTAGGAAAAATTGAATGGTCAAAAAATGTTAGGGATGCTTGGGAAGCTTTCTGAAAG |
| TGAGTATGTGGAGGAGGGGTGAAGAATTAGGGGTTCACAAGAGAAGTATTTCTTTTAAGGAGTGTATAAAGGA |
| AAGATATTCAAAAGGAAATATATTCAAAAGGAAAGATATTCAAAATCTGCAAGTTCTTTATGCCATACAGATT |
| TTGGGGACGTTTACAATTTCAACAATCTTGAAACAGTTTTCAAGGTTTGGGTTTTTTTTTTTCTTTCCTTTTTT |
| TCTTTCTTTTTATGACAGGTAAGGTGCTCAGGTCAGAACAAAGTTTGAGGGAGAGACACATCTCACATATAAG |
| CGTGAAACCCCAATCATCACACTTGTGAACTACAAAAGGGTCTCTCTTTTTTTTGAGACAGGGTCTTGCTATG |
| TTGCCTTGGCTGGAACTTGACCTCCTGGGCTCAAGTGATCCTCCCCTCTCGCCACCTGAGTGGCTGGGACTAC |
| AGGCACGTGCCACTGAGCCCCACTGGAAGTGGAATTTGATATTCAGAATTGTCTTTACTTTTCAAGGATCCAG |
| TCCATGTAGTGACTGGTCACATCTTCCTCTAAAACAAGTGCAAAGTAGTTAACATCTCTATATAACAAGAAAG |
| ATACCAAGCCCCAACCGGTGACATTTAATTAAAGAAATGTTGTATTTATGACATTGCATACAGCATAGAATTA |
| GGTAATTCATTACAGTTTCTGGTAACCTATTTAAGATGCATCTGAATAAGTCTCTTTTTTCATTTTTCCATTT |

TTGTAAATGGAGAGCAGCTCTGTAAATGTTCAAACTGTCATAAACTCAGGTATTTTTACTTTCGAGTGCATTA
TATATTTCTGTAGCCTATTTATATTCTAAACTAACTTATCTTACTACTAATTAATTGTGGTGTGAGTTTAACT
GCAGCAATGCTAGCAGGGTGTGAGGCTTAGGAGGCCTGGAAATGGCTAGGAGCAAGACTGCTAGCTTAAACTT
GAAACTTTTTGGAGTGTGTAGTTTACATTTTTCCTCTTCTTCTTCTATTTAAACTCACATGCTAAACCAGCTG
GAGGTCAAATTTTAATTTTATTTGGAA**GCCTGGCTAAAAGGATAATCTAATTTTTGCTGCTTTTAGAATTTGA**
**TGCTAAATTTATCTTTGAAGAATCTCATTTGTTGTTTGTAAAGGACTCTTTTTGAGATGGAGCCTCACTCTGT**
**CACCCAGGCTAGAGTGCAATGGCGTGATCTCAGCTCGCTGCACCCTCCGCCTCCTGGGTTCAAGTGATTCTCC**
**TGCCTCAGCATCCTGAGTAGCTGGGATTACAGGCTCCCGCCACGATGCCCAGCTAATTTTTGTATTTTTAATA**
**GAGATGAGGTTTCACCATGTTGGCCAGGCTGGTCTTGAACTCCTGACCTCAGGTGAGCCAGCCACCTTGGCCT**
**CCCAAAGTGCTGGGATTACAGGTGTGAGCCATCACACCTGGCC**TGTAAAGGACTCTTTAACAAAGTGTTCTTA
GGTGTGACAGTGGTGACTTCATATTACAAACTGGAAACAGGGATGTGGAGTGTTTGTGTGTAAAACAGATTGT
GTGTGTGTGTGTGTGTGTGTGTGTGTGTATAAAACATATATATAGTGTGTATATATAGATATATATTGTTTAG
ACTATATATATATATAAAAAATCTAAACAAAGATAGCAAAACTAAATGAAATGTAGTGACCACTCAACTTTAC
TAAAAATAATCACATGAAAGTAGTGTTGCCCTGGAGACTCATGTCATCGTTCTGCTCTAATGAGTCCTTGTTA
ATGACCCAAATTTTAATAGAGTAGCCTTAGTAGCCTTTTCTACTCAAGAGACCTTCATATCTAACCCAGTTCG
GCAACTCACTTTACTGGCCGTATCCTGCAGTTTGTCATGCAAGCCTGACTTCTTCCACTTCCTGAATTCTAGG
ATAACAGAGTTCAGCTACAACCTACGTTTTGTTCACCTCCCTCATTCTAGTACTCATAAGTCAGTGCTCTACC
TAGTCCAAGCTTTTGTGGTACCACGGCCCCCTTTCCCTTTCTCCAGTATATTAGCCCCCTCATTGTTTTGCCT
TTCTTCCTGCAATCTTTACTCCACTATTGGCCTTTTCGGTGCCTCTCAATTCCCAGAGAAAGTCATACTCATG
CTAGTAGATAATATTGCAAATCAAAGGTTTCTAACCTCAGACATTCGCATCCGGTATCTTTTTACTTTCCCTG
GCCATCTCCTTTCCCTATGGGAACTATATAAATAGTCACCATTCATGTCCCCATTATGCTTAATATGTGACAT
TGCTTTTTTCTCACACAGGGAAAATAAAAGTTTATCAAGCACGGATTTCCTCATTTTCCTGTCTTTTGAAGTT
ATGTGTCCCATCCATTACTGCCTTCTTCCCATCTAAGAGGAAAAGTTGTTCAAGAGTGCAATACTCAGTCCTT
ACAATTTTGTTCCCTGTCATATGCCTCCATCCGTTATTCTCTTTCTATCCTGTGTGTTTAACTTCTCCCTTTT
CCCTGTTGCCTTCACTGTTGCCCCCACACAAGCTCAATCTCTCCTATCCTGAAAACAACATAAAATTTGACCT
GGCATCTACTGGATACCACCTTGTGCCTCTGTGTTCTTCAGTCCACTGCAATGCAGCTCCTGCTCTCACTACT
TAGTTGGAATCACTCAAGCTGTGACCACTGATGACTTCTTAAATTGTCCAGTCTATCGTTCTCTTTCCTCCCA
CATCTCTTTTGAAACTTTTGACTTTATTAACCACACCTTTTAGAAATGTTTCCCCTTTGGCTTTTGGGACTAC
AAGCTCTTTTTATTTCTTTCTCTGTCTCTCTAACCATTTTTTCTTAGTTTCAGTGACTTGAAGTTTTGCTTTT
TTCCCTGTTCTCTCCATGGGTCTGATGTTGTCCTACTTCTCTACAAATACTGTGCACTCTCCATATATGATTT
ATGTATTTTCACAGCTTCAGCTATTATCTGTAGAAAGAGACTCCCAAATCTTTATTTAAGCTAGGTTCTCCCT
ACTGAGTTCTAGACTTAGGTTTTAGCTGCCTTACTGCTCATCTCCCAGAAATCTCAGCTGTAGGCATCAATAA
ATAACCTTGTACTCTTTCTTCTAGCTTACCAAACCTTATTTTGCCTTATGAGTGCCTCCTGTATCCCACAGTC
ACTGTCTTAGTTCAGGTCTCATTATCTTTCATGTGGTCTCTTGCAGAAGTTTCATTCTTGGCTGCATGCTCAC
TCCGGTTTGTACTTTTAACTGATATTTATTCAGCGATATTGATTAATTACCTAATATGTACCAGGCCCCATGC
TGGGCTCTGAAATATAGTGATGAGTGGAAACAGGCATGATCCCAGTTCTAATGGAGCTTACTTTCTAATGGGC
GAGACAGAAATTATTAATATAATCAACCAGAGTATGCCTTTGAAGGAAAGGAACACAGTTCTCTGAGATAATC
TAACAAAGGAAATGACCTACTCTAGGAAGACAGGGAGACTTCCTTGAAAAGGTGTAATGCAACTGAGATCTGA
TGGGAAATATTTTCCGACCGTATTTTAGATAGAACAGCATGTGCAGAGACCCTGTGGTAGAAGGTGCTGCAGA
GGCAGAGAGAGGAGTCAGACCATAGAGTCCTATGGTCACATTCTCCTAGAAAGATGGGAATCTAGTGAAGGAT
TTTTAGCAGGTAGGGAGGGGGATGTGGTAACATGATGAGAAAATCATTCTAGTTGCAATATGGAGAATGGATC
TGAAGGGGCCTGGGATACCACTTAGCAGGTCACAGAGAGCAATCCTTCTAAAAGGCATAAATGAACTTTCCCG
TCCTCAACATTCTCCACTTGTGCGAAAGTGAAATCTAAACTACTTAGCTCAAAAAACAGAGTCCTTTCTAGCA
CTCAATAATGTTTCTAGTCTTTTCTAACGCTTCCTGATGTGCATCCTGTGCCTGGCCTTGTGTTCACTGCTAC
TTGATCTTGCCACGCTCTGTTTCTTCTATCTGTACTGTCCTCTTCATCCCCCTTTGTGAACCTAGGGAACACC
TATTTTCCAGCAAAACACAGCTCCTTCATTTCATTCCACCCCAGAAAAAAAACCAACAAAGCTCATTGTTTAT
CCCAAGTACCCACAGTGCAAGCTATTGGCACTTATTCTACAGTATTATAACAGTTTATTTACCTGTACTTTTC
ACACCACGGAGCTCCTTGTCATTAGGGATTATTTTATCCATCTTCATCTCGTCCTTTTTTGTCTAGCACTTTG
CCTGGTGCCTGTAAAGATTGGGTAAGCATTCCATTTTTCTGCTCTGACCAGTTTACTTTTACCAGTAGTCAGG
TCAGTTAAACAGTTATTGACCACTTAACTCTGCTGGGTGTGTGGGATACTAACACACCCACACCACAGACCCT
ATATTTGATCTGCTTTTCTCATGCTAACTGTGCTTCTCTTTATTTTATTAATTTACACTTGAAATTTGTCTTA
ATAGGAACTTTTTATTCATTGTATAGGGCTAGAGTAGAGAATCATCATTTGTCAAATAACTGTTTTCTGGAAC
TGCAGAAAATGTCTTTTGAAATCTTAAAATTAAGGATGAATTACACAATTTTACCTAAACCGCAGAAGAGTAC
TCTAGTGAATTACATTAGCTCCTTGGCAATCAGTAATTTCCATTGTGTCTGTCAGGATTCCACAGGAACGAGG
AGGCCCTTGGTGCTTCGATTTAGGGACAGATTTTATAATGTTTTTGCCCCGATCTGTTATTTGGGAGAGGGTC
ACAAATTGACTTGATCTTTGTTTTTCCTTCTTTGTAG

**Intron 6, Sequence 5'→ 3'**

GTAAGCTGGGATTTTGTAGTGTTAAAAAATGTCTTGTCATTTTTTTTTTTTGAAAAATTTTTCTAAAATTAAGT
ATTTTCTTACATGTTTGCCCTTCCTCCATTCTTCTTACCTCAACTCTTACCCCATCATTCTCCCACCAACCCT
CTTTCCACAAAGCCTAAAATCCTTAAGCATTTCTTGAAAAAAAAAAAAAAAAAAACCTTCAGTCAGTATTTTAG
CTTAGATTTTTTACATTTGATATGTTAATGGCTTGTCTTAAAATTATGTTAGTGGTAATGTAAATTAAAATTT
AAACTAATTTTTAAATGTAATATTTGTTTTATCTTACTGATATTCTTAGCAAATTATTTCACAAGTGAGCCAA

AAAGGATTAGTCACATTACAACTAACAGTTCTGTGTGTAAGTCCTAAAGCAAAATTCAGTATTGGAAAAAAAA
GTAAAATATATTTAAATATTCACCAGCAAAGCCTCTCTTGAAATGGGTAGAGCATTCCCTTTTTAGCATGTGG
AAAGGATATTTGTTTGTTTTATTTGAACAAATAAGTAAACTGCTGGAGCACTGCAAAATCCTCCTTCCTAGTT
TTACCCTCAGTGCTTCTGAAGTCGGGTTCCATGCTATCACAAAAGTCGTTTTCTTTACCCTCTCCCTTCTGAA
ACCTGTATTGGCTCCCTATTACTTTAAACTAGAGTAATGCTGCTCAACTGTGCCCTGAGAGTTTCTCCCATCG
CCACCATCTTTTCTCGGGGACCTCTTTCTCTGCTTACTCCTTATGCTTCATGCACACTGATAGTCTCTAGTTT
TGTAATCCCAGCACATCACTTCTTTTTCCTGGAACACCTCTTCCCTCGGATAAATTAATCAAGTACTTTCATT
TCCCTTTTTTCTGGGAGTTTTTTTTTTTTTTTTAATTCTCATATATGAAATCCTATGTGGGATTTTACTTCAA
AATAATACCAGGTGAAGGAAAGTGAAGAGGGATATAGATGAAACAAGTTTGATCGTTTATTAATAATGATTGA
GTTACGGGTATGTCAGAATCTTTTCTGTCTGTTGTGTATATGTTTACATTTTTCCGCATTTAAAATTTCTTTT
TTTTTTTTTTAATCCCCTTTGAGAAACCTGGAGTGAAAGTGTGACAGCCAGTCTGGTGACTAGTTTATCTTTG
CCTACTCAAAAGCCATAAAACACTATTTTTATGTGTGCTACAAATAGTTAATTCATGGACTAGGCACTTGATT
TCCTACTGTTTCGTGTCTGTGTTCTGTGTATCTGTCGTTACTCATGTTGTCTGCTAGGATTAGAAAACAGCAC
CAAATTAAAGGCCATATGCTATGGTACTTAAGTTCTTTTTATGCTGATTTCTTTTATAAAGTAGATTGTTTCT
CCAGCTATATTGATAAGGAATAGGATGAAGACCTTTTATTTTTAAACCCTTGATTTAGAACTGTCTTCAGATA
AAATTGTTAGGCCCAGATTCCAGTAGCCCTTCCATGTTTAGTAAGCTCCTAATGTGAATCATAGATATTGTGG
AGCTGTACTGTCCAAAATGCTAACTACTAGTCACATCTACATGAACTTAAAAAACAAAAACGAAATTGACTTT
TTTAGCCCCAGTAGCCACATTTCAAGTGCTTATTAGCTACTGTGGCTAGTATGTACCATTTTGAACAGAGGAG
ATATGGAACATTTCCATGATTGCAGAAAGTTCTATTGGACAGCGCTACTGAGAGTATCATAGTTTCTTGATGT
CTTAGTAGCATGGCTCAGATATTTAAAGCCTTCTTAAGGTATTTGTATACTCATGAACAAATACCTCTGGATT
TCATTATTAATCCATGTCTATATCTTCTTCATTGACTATGGTAAGTAAGAAGGGACATTTCAAAGTAATTGTT
CATTTTAGTTCCTGAAGCTCTTTTCTTATCAGTTCCTGCCAGATGGCACTTCTCCAGAAATGCAGTCTAATTT
TTATGGAATAATTTGCATAACTCAGAACTTAACTGAAACTATTTTTAACACCTGCATATGACAGTTGAAAGTA
ACTGTTTCAAGTAATTAACATAATTTGAAAGTAGCAGCTGCATATGTGTTAAACAGTTTCTTAAAATTTACTC
TATGAAAATTATTTTTGGAACTCTTTACTGGAAGAAACTAGTACAGTGATTGTTTTGAACCAATCATACAGAC
AAATAGGAAAGAAAGGAACGCTGTTAGTAGCAGTTCAAGAGGTGTGTTAGTCACAAATAACTTAGTTTCTTTC
AAAGATAAAACTTAAATTACATATACTGTGTATATAAACAGTTCTCCATTTTGCCCATGAGAAGATTCAAAAT
ATTTCGTAGACAGCTTGAATATATGAGCTTTTAGGGAAGTCATGAAATAGCCAAATGTTTTATTTCTAATATT
CAATTTTAGTTTAATTATACATACATATAATTATACATACCTATAATTGCATATCATATAAAGTATTAGAAAT
AGTATTTTCCCCCTTCCTACTTCATGAAAGTTTCATTACACTTTTCATATGCAACTTTGATGTTAGTATCTGT
CATTCATCATCACTAGAACCACTTTTTTTTAATCTGATGACATTTTTTCCACATCATGACGTCATCTTCTGTC
TCACTGGTTCCTTCATTAGACCTTATAAACCTTTAAAAATGACATTGATTGAAGTTTTCGGGTGTATGTGTCT
TACGCAAACATTTATTTGTCAAGATAATTAATACAGAGCTCTATAATGCAGAGCTTTGTAAAGATTTAATTAT
AAGATGTACCATCTGGGGTTTGGGGGGAGATCTTTGCCTTATATTTGGGGCATATATTTGATATTCAGCTTTG
TTTATTTTTCAGTATAGAGTAGTCAAGAAATGTGTTTTTTATAAAAATAAATTTTTTGAAGTATGTTATATTT
TTTAATAAACAGATGTTGGAATAACAACTGAAAATGGCACTTGAAGTTATTTACGATTTTATAATTTGTTATG
ATTTCACCCAGCCTTCTCTGTTCTCTCCCTTCCCCCACAACCCATGTAAATGTGTACGTCCAGAGACCATTGT
GCTGGTGCTAGGCAAGGCTTTGTTGTCGGCCTCAGACATGTGAACACCGTGCCATCGGGGCTGTAACCCCAGA
GGAAATGGAGAACATTGAGGGAGGCTGGCCTACCACAGGAATACTAAAAAGTAGCCATTTTCTAGATAAAATT
TATCATTTTAAAAATCATGGTAAGCCAGGCATGGTGGCACACACCTGTAGTCCTGGCTACTGGGGAGGCTGAG
GCAAGGGGATGGCTTGATTCCAGGAGTTTGAGACCAGCCTGGGCAATGTAATGAGACTCATCTCTAAAAATAA
AATAAAACTCATGGTACACTACCCCTACTTCCATGTATTTTTTGTAATCATTTGGGAATACCTTTATGTAGGG
TGTTTGTTAAGTTAGCCACAATTCCTACCACCCCTTGTGGTATTCTACTCATCTCTTTTTTTTTTTTATTATT
ATTGTTGTTGTTATCCCCATTATACAGAGAGATTAATTAGCTTGCCTAAGGTCCCTCAGCTAGTAAGTGACAC
AAGTATTTACCCATTCTCTTACTAATTTACTTTTTCTGTGGTAAGCTTTGAGTGTACAAACACTGCTTTAGAG
ACACTAGTCAATTTGTGCAAAGCTGAATGGACCATTACTTAGAAAAAGGTTTCTTGATAATCCTTTTTGAAAG
CAATAGATACTATTTTTAAAACTTTTGCATAGGCATTTGATGGTTGTGAAGATAGAACCTAGTGATGGCATTT
TACCCTCTATTATTATTGACTCGGTTGACTATCCAAAAGGAAAGGCTACTGGGCACAGTGGTGCATGCCTGTA
GTCCCAGTTACTCAGGAGGCTGAGATAGCAGGATCACTTGAGCCCTGGAGTTGGAGGCTACAGTGCAGCATAA
TTGCACCTGTGAATAACCACTGCACTCCAGCCTGAGTAACATAGTGAGACCATGTTTCTATTTTTTAAAAAGG
AGGTATGGGGCTAAGATACAAAGTTGAATCTGAACTTAGGAAAGAAACTCTATGGATTTTTCCTTAGCTAATG
TCAAGCACAACTGCGCTCCACCTAATTGGGTTGAAATTTAAAGACAAAAGATCAGAAATGGAATGGCTTTTTG
AATTATAGTTTGTTTGTAATTTATAATAGTGTTTTTAGTCTTGTCAGAGAAGAAAAGAGTAGGCAGCTTAGGT
TCTCTGCTAATTGACCAGTGTGAATTTTAAAAACTTACTTGTATTTCTTTTAAATTAAAAAAAAAAAAAATCCT
CCTAAGTAGTTACATTACATTATAGTTTTTCCAGCAAATTAGTTTTTTTTTTTTTTTTACATTGCCTTACCCAA
GTTTTTATGCAATGAAATGAGTTCAATCAGCAGTTAAAAACTAAAACGCTTCCCTCATAAGTTTTAAGGGTTT
TCTTGGTCTGTTTTCTCCTCTGAAATGTGGAGCATTGAGTAACACATATGAAAGAATATGGCAATATTTTTTA
TACCTAGTCATTTATGTGCCACCTTCCTGCTTTACCATATTCAC**ATACTACTAATTCATACTTTTGACATTTT**
**AATTATTAAGGTATATTAAATAACAAAATATTTTGGGTTTTTGTTTTGTTTTGTTTTGTTTTGAGACAAGGTC**
**TTGCTCTATCACCCATGCTGGAGTGCAGTGGCCACAATCACGGCTCAGTGCAGCTGCAGCCTCAACCTGCCAG**
**GCTTAAGCAATCCTACTACTTCAGCCTCCCAAGTGGCTGGAAGTACAGGCATGCACCACTATGCTTGGCTAAT**
**TTTTTGTATTTTTTTAGAGATGGGGTTTCGCCATGTTGCCAAGGCTGGTTTCGAACTCCTGGGCTCAAGCAAT**

**CCACCTGCATCAGCCTCCCAAAGGGCTGTTATTACAGGCGTGATCCACCAC**TTTATTTAAAATTTTAGTTACA
GTTAAATAAAAGTATTTCATTGTGTACTGTCTGCAATCATTTTGTTCAGTACCAATGTACACACCACACTGGG
AAACACTGGCATATATATAGTGGCACTTCTACATGCATTGTACATCAAATTGCATTGATAATACTAAGTGAGC
TTTTCTGAGATGATTATAACGGGAGAAGAGAGGTTCCCTTGTGGTTGCATGTTATCTCAACACCCTTTCTCCT
TCCTTTTGTAAGCTGAAATATTTCATTGGAAATTTACAAATTATGTAGAGAGGATCTTAAGACCGTCTATTTT
CAGAGATACTTCTCTCTTCCATTGGAAGAATGACTGTAAACTTGGTATCAATTTTCCTAAGAGTCCCAAAATA
CAGTACTGTAAATTGTAATATTCAGACTGTCTTATGTTCTACCTAAAACTTGATCTTTGCGCCATGCTTACTA
GAGAAGAGTTTTCCATTTCAAAACTAGACTTCAAAAAATCTCTACTCCTCAAAAGATTTGGTGAAGTAGCTCT
CAGTACTGAAACACAGAGAAATGGAGGACCAGTAGACCTGCACTCAACTGTGCTAAATGCAAGAGATAAGTTT
AGTGAGGCTGCAGAGGAACACAGTTCAGCCTGCCAGCAGCAACCCACCATTTCCATCGTTCTAATTAGTAGCT
GTAGAACATGAATAGTACTAACTAGACTAATATAATCCTTGTAAATATACATACGAAGAAAACTGCTACAACA
AAGGAAATTAGAAATGAGGTATAGGTGTTTCATTAGTCAGTAGGCTGGCCTGTTGAGCTTATCACTGTCAGTG
TTTTGTAAACATTTTTACTGCAACCAAAGATGAAAAACTTTTTACATCATAATTCATTGTGTGTTTGTGTGTG
TGTAAAATGTGGAATACA**ATTTTCATAATACAATGTGCTATATACTCTAGCATTTTCTTTTTCTTTTTTTTTC**
**TTTTTGAGACAGCGTCTGGCTCTGCCGCCCAGGCTGGAGTGCAGTCACAATCCTAGCTCACTGCATCCTTGAA**
**CTCCTGGGCTCAAGCAATCTTTCTGCCTCAGCTTCCCAAATAGCTAGGACTACAGGCACATGCCACCATGCCT**
**GGCTAATTTTTAAAACATTTTTTTGTAGAGATGGGGTCTCTCTCTGTTGCCCAGGCTAGACTCAAACGCCTGG**
**CTTCAAGCAATCCTCCTGTCTCAGCCTCCCAAAGTGCTGGGATGACAAGTGTGAGTCATGGTGCCTGGCCACA**
**TTTTCTATTTTATTTCATGTTTTAAAAGATGCTGGTGTGACCCCTAAATTGATTTCATGATCCACTAGTAGGT**
**CTTTTTTTTAAAAGACAGAGTCTTGCTCTGTCACCCAAGCTGGAGTACAGTGGCATGATCATAGCTTACTGTA**
**ACCTCGAACTCTTGTGTTCAAGTGATGCTCCTCCTGTGTCTGCCTCCTGAGTAGCTGGGACTACAGTTGCATA**
**CCACCACGCCTAGCTAATTGTAGTTTTTGTAGAGACAGGGTCTTGCTGTGTTGCCCAGGTTGATCTCGAACTC**
**CTGGCCTCAAATGATCCTCCCACCTCGGCCTCCCAGAGTGCTGGGATTATAGGCATGAACCATCACACCTGGC**
**C**CCACTAGTGGGTCTTGACTTGTGTTTTCTAAAAATACTGTTTCTGAGATAACCAGCTCAACTAGATAACTGT
GGGAGAAAAAAAGAAGTAAAAATAAAATCTAGGTACTTGGGCTTGCAAAGTAATTGAGGAAAAAGAATATACA
ATTAAGGAATAGTATAAATGAGAATACAAAATGTTAAACTTGTTGTAATGACTGACAAGGGACTAAAGCATGA
GCTGAAAATGTGAATCCTATTTTAGGTAAACAGAAGTGTCTCTACTACTATGAACATAACAATTTTAATATTA
GAGGAACATCAAATTGTCAGCATGTAACTATGATTAATATTGTGTCATTTAATACACATTAGAAAAAAGAGCC
ATAGATCTTAGCTCTAAAAGGGATTTTGAGTTTATCTGATTGAAGCAAATCTAAGTATTCACATTTTATGGAT
GAAATAACTGAAGCCAGCAGAGGTTAAGTGACTTGCTAATGCTATATACCTAATTGAGGATGAGAGATTGCGG
TGGGGTGGGAGCAGAATAGCTTTCAGATCTCCTACTTCTACATTTTTCACTTACTCTTAAAGAACAGAAATGA
GCCAAGCACAGTGGGTCACGCCTGAAATCCTAACCCTTTGGAAGGCCAAGGTTGGGGGTTCTCTTGAGTCCAA
GGTTTTGAGACCAACCTGGGCAACATAGCAAGACCACACCTCTTAAAAAAAGGGAAAGGAAATGTTTTAGCAT
GTTTTAATCTATAATGTATTTTTTTACAG

**Inverse complementary sequences, sequence match 1**

70.2% identity (70.2% similar) in 389 nt overlap (4262-4642:6195-6571)

Intron 1:
GCCTGGCTAAAAGGATAATCTAATTTTTGCTGCTTTTAGAATTTGATGCTAAATTTATCTTTGAAGAATCTCA
TTTGTTGTTTGTAAAGGACTCTTTTTGAGATGGAGCCTCACTCTGTCACCCAGGCTAGAGTGCAATGGCGTGA
TCTCAGCTCGCTGCACCCTCCGCCTCCTGGGTTCAAGTGATTCTCCTGCCTCAGCATCCTGAGTAGCTGGGAT
TACAGGCTCCCGCCACGATGCCCAGCTAATTTTTGTATTTTTAATAGAGATGAGGTTTCACCATGTTGGCCAG
GCTGGTCTTGAACTCCTGACCTCAGGTGAGCCAGCCACCTTGGCCTCCCAAAGTGCTGGGATTACAGGTGTGA
GCCATCACACCTGGCC

Intron 6:
GCCTGGCCACATTTTCTATTTTATTTCATGTTTTAAAAGATGCTGGTGTGACCCCTAAATTGATTTCATGATC
CACTAGTAGGTCTTTTTTTTAAAAGACAGAGTCTTGCTCTGTCACCCAAGCTGGAGTACAGTGGCATGATCAT
AGCTTACTGTAACCTCGAACTCTTGTGTTCAAGTGATGCTCCTCCTGTGTCTGCCTCCTGAGTAGCTGGGACT
ACAGTTGCATACCACCACGCCTAGCTAATTGTAGTTTTTGTAGAGACAGGGTCTTGCTGTGTTGCCCAGGTTG
ATCTCGAACTCCTGGCCTCAAATGATCCTCCCACCTCGGCCTCCCAGAGTGCTGGGATTATAGGCATGAACCA
TCACACCTGGCC

**Inverse complementary sequences, sequence match 2**

68.8% identity (68.8% similar) in 346 nt overlap (4301-4642:5859-6202)

Intron 1:
AATTTGATGCTAAATTTATCTTTGAAGAATCTCATTTGTTGTTTGTAAAGGACTCTTTTTGAGATGGAGCCTC
ACTCTGTCACCCAGGCTAGAGTGCAATGGCGTGATCTCAGCTCGCTGCACCCTCCGCCTCCTGGGTTCAAGTG
ATTCTCCTGCCTCAGCATCCTGAGTAGCTGGGATTACAGGCTCCCGCCACGATGCCCAGCTAATTTTTGTATT
TTTAATAGAGATGAGGTTTCACCATGTTGGCCAGGCTGGTCTTGAACTCCTGACCTCAGGTGAGCCAGCCACC
TTGGCCTCCCAAAGTGCTGGGATTACAGGTGTGAGCCATCACACCTGGCC

Intron 6:
ATTTTCATAATACAATGTGCTATATACTCTAGCATTTTCTTTTTCTTTTTTTTTCTTTTTGAGACAGCGTCTG
GCTCTGCCGCCCAGGCTGGAGTGCAGTCACAATCCTAGCTCACTGCATCCTTGAACTCCTGGGCTCAAGCAAT
CTTTCTGCCTCAGCTTCCCAAATAGCTAGGACTACAGGCACATGCCACCATGCCTGGCTAATTTTTAAAACAT
TTTTTTGTAGAGATGGGGTCTCTCTCTGTTGCCCAGGCTAGACTCAAACGCCTGGCTTCAAGCAATCCTCCTG
TCTCAGCCTCCCAAAGTGCTGGGATGACAAGTGTGAGTCATGGTGCCTGGCC

**Inverse complementary sequences, sequence match 3**

68.3% identity (68.3% similar) in 375 nt overlap (4276-4634:4571-4942)

Intron 1:
ATAATCTAATTTTTGCTGCTTTTAGAATTTGATGCTAAATTTATCTTTGAAGAATCTCATTTGTTGTTTGTAA
AGGACTCTTTTTGAGATGGAGCCTCACTCTGTCACCCAGGCTAGAGTGCAATGGCGTGATCTCAGCTCGCTGC
ACCCTCCGCCTCCTGGGTTCAAGTGATTCTCCTGCCTCAGCATCCTGAGTAGCTGGGATTACAGGCTCCCGCC
ACGATGCCCAGCTAATTTTTGTATTTTTAATAGAGATGAGGTTTCACCATGTTGGCCAGGCTGGTCTTGAACT
CCTGACCTCAGGTGAGCCAGCCACCTTGGCCTCCCAAAGTGCTGGGATTACAGGTGTGAGCCATCAC

Intron 6:
ATACTACTAATTCATACTTTTGACATTTTAATTATTAAGGTATATTAAATAACAAAATATTTTGGGTTTTTGT
TTTGTTTTGTTTTGTTTTGAGACAAGGTCTTGCTCTATCACCCATGCTGGAGTGCAGTGGCCACAATCACGGC
TCAGTGCAGCTGCAGCCTCAACCTGCCAGGCTTAAGCAATCCTACTACTTCAGCCTCCCAAGTGGCTGGAAGT
ACAGGCATGCACCACTATGCTTGGCTAATTTTTTGTATTTTTTTAGAGATGGGGTTTCGCCATGTTGCCAAGG
CTGGTTTCGAACTCCTGGGCTCAAGCAATCCACCTGCATCAGCCTCCCAAAGGGCTGTTATTACAGGCGTGAT
CCACCAC

**Supplementary Table 5: Primer pairs and length of PCR amplicons.**

| Transcript | 1. forward Primer 5'→ 3'<br>2. reverse Primer 5'→ 3' | Amplicon length (bp) |
|---|---|---|
| 18S rRNA | 1. CACATCCAAGGAAGGCAGCAG<br>2. GACTTGCCCTCCAATGGATCC | 152 |
| TRAM1 (convergent) | 1. ATCTGGTCAGCTTAGTGCGT<br>2. TGGGGATAAGCCCTCCATAAGA | 111 |
| TRAM1 (divergent) | 1. GTTTGGGGCACATTCATTCTCATC<br>2. GGAGAAGTGCATTCGCCTGT | 113, 320, 363, 384 |
| circTRAM1-56 | 1. CTACTTCCAGAAAACCAAAAAAAAGA<br>2. ACAGTAGCCAAATCTTTGATGCC | 83 |
| circTRAM1-57 | 1. CAGCTGGCTTACTGGCTTCA<br>2. TTGCTGGGAGGGTGACATTG | 118 |

**Supplementary Table 6: Antisense oligonucleotides (asON).**

| asON | Sequence 5'→ 3' | target transcripts |
|---|---|---|
| asON linE4-1 | GTAGAACGCACTAAGCTGACC | linear and circular TRAM1 RNAs |
| asON 56-6 | TCAGTAGCTTGTTCTTTTTTT | circTRAM1-56 |

## Supplementary Figures

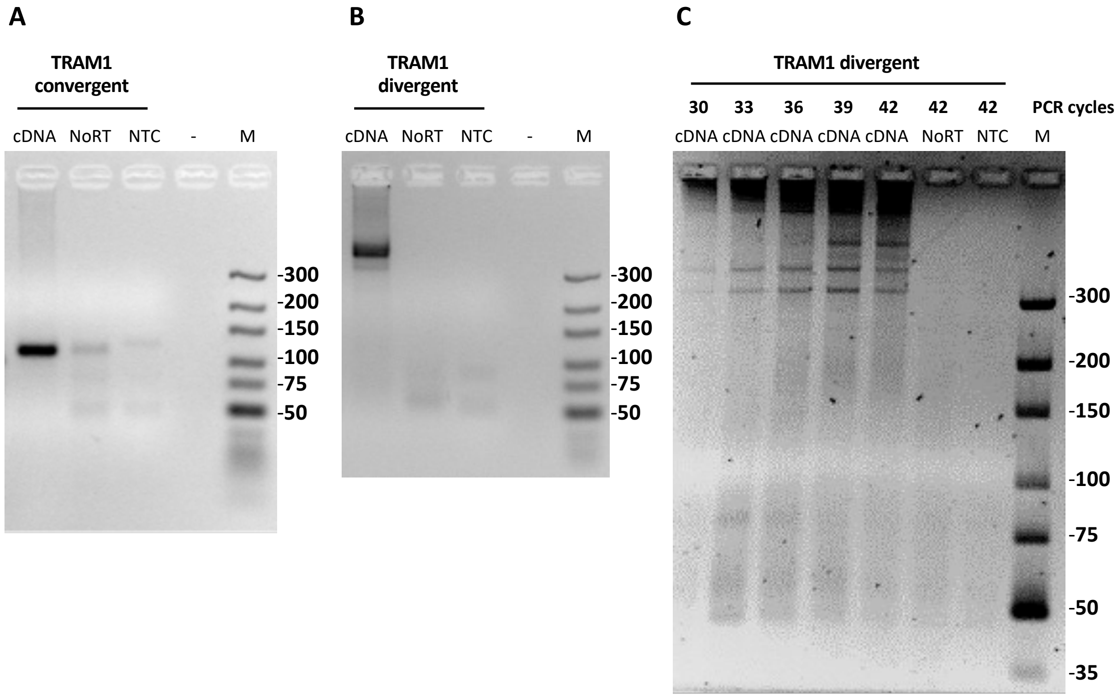


**Supplementary Figure 1: Figure 4 full-length gels.** PCR-based detection of linear and circular TRAM1 transcripts.

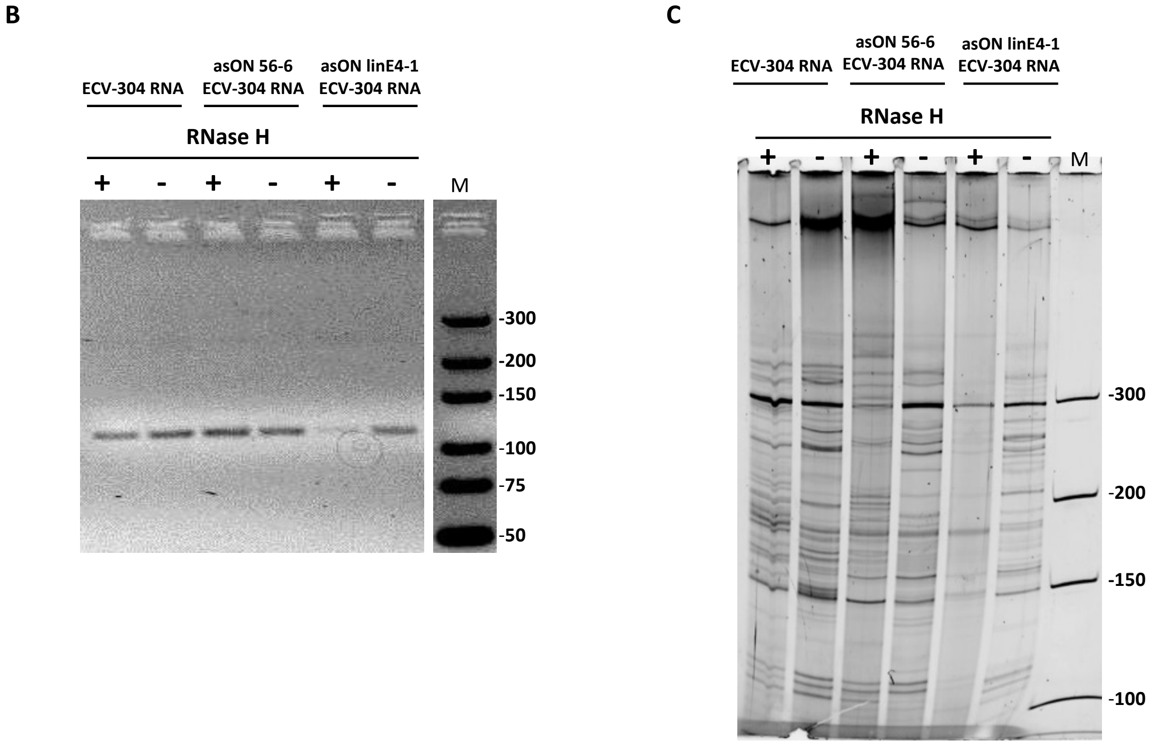


**Supplementary Figure 2: Figure 6 full-length gels.** RNase H treatment of complexes between cellular RNA and TRAM1-specific asON indicate circular topology.

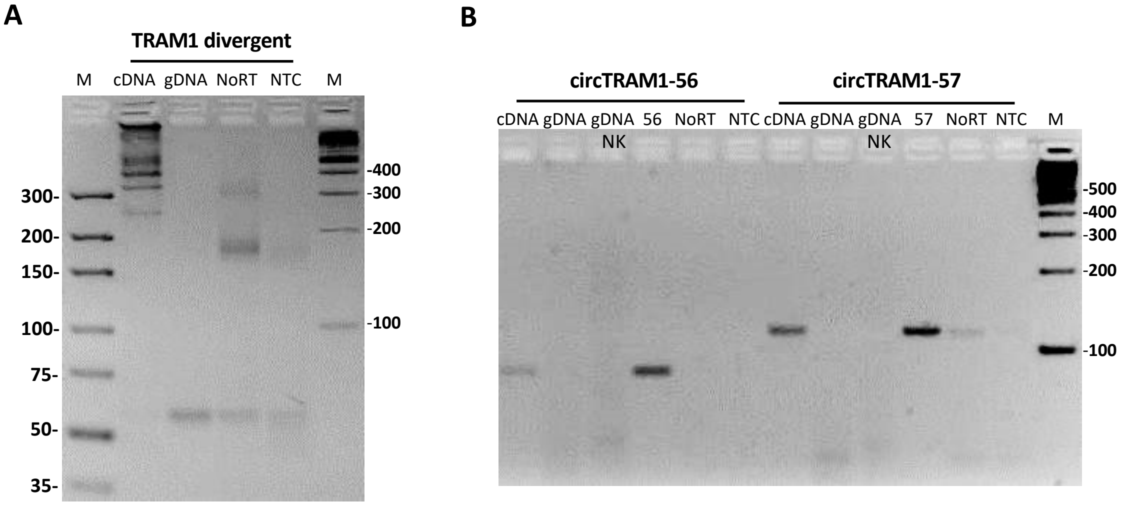


**Supplementary Figure 3: Figure 8 full-length gels.** Examination of the tandem repeat hypothesis for circular TRAM1 transcripts.

**Supplementary Methods**

**RNA Isolation from urine samples:** Spontaneously voided urine of donors was collected and stabilized with one volume of a lysis buffer (6 mol/l guanidinium isothiocyanate, 0.05 mol/l sodium acetate, and 0.5% N-lauroylsarcosine) as previously described [38]. Stabilized urine samples were frozen in liquid nitrogen and stored at -80°C. To isolate RNA from human urine, stabilized samples were thawed slowly and adjusted to pH 7.0 by adding 1 M HEPES buffer. RNeasy Midi Kit (QIAGEN, Hilden, Germany) was used for RNA preparation with minor modifications: Instead of using RLT buffer, recommended volumes of 70% ethanol and mercaptoethanol were added directly to the samples. Subsequent steps were performed following the manufacturer's instructions. RNA samples were eluted in 320 µl of RNase-free water, lyophylized, and re-suspended in 16 µl of RNase-free water. Urine RNA was stored at -80 °C.

**RNA quantification and quality assessment:** RNA samples were quantified using a NanoDrop ND-1000 spectrophotometer (Thermo Fisher Scientific, Waltham, MA, U.S.A.). Agilent 2100 Bioanalyzer in combination with the Agilent RNA 6000 Pico Kit (Agilent Technologies, Santa Clara, CA, U.S.A.) were used to measure concentration and integrity of pooled urinary RNA.

**Synthesis, purification and quantification of double-stranded cDNA libraries:** Seven urine samples were pooled and used to compose the final patient pools C and HR (Supplementary Table 1). Both urine RNA pools were used to produce cDNA libraries which were synthesized using the SMARTer Stranded Total RNA-Seq Kit – Pico Input Mammalian (TaKaRa Bio Inc., Kusatsu, prefecture Shiga, Japan). According to manufacturer's instructions, 10 ng of urine RNA was fragmented and used for first-strand synthesis. For sequencing on Illumina platforms, Illumina adapters were added and ribosomal cDNA were depleted. The final double-stranded cDNA library was amplified via PCR according to manufacturer's instructions and purified using the Agencourt AMPure XP PCR purification system (Beckman Coulter, Brea, CA, U.S.A.). The first purification step was performed according to the manufacturer´s instructions. The second purification step contained minor modifications: The amount of beads was increased to 100 µl volume and the cDNA was eluted in 20 µl elution buffer. Quantification of double-stranded cDNA libraries was performed using the Qubit dsDNA HS Assay Kit and the Qubit Fluorometer (Thermo Fisher Scientific, Waltham, MA, U.S.A.).

**RNA sequencing:** Double-stranded cDNA libraries of patient pools C and HR were sequenced by GATC Biotech AG (GATC Biotech AG, Konstanz, Germany) using HiSeq4000 (Illumina, San Diego, CA, U.S.A). The Illumina platform was used with paired-end (PE) mode and read lengths of 125 nucleotides.

**Data quality control:** Quality of reads provided by GATC Biotech AG was checked using the software FastQC (https://www.bioinformatics.babraham.ac.uk/projects/fastqc/) by Simon Andrews at Babraham Bioinformatics.

**Data analysis:** Codes for analyses of transcriptome data were written in the UNIX command line. First, the cDNA and ncRNA databases provided by Ensembl (version e87) and the circRNA database provided by circBase (version updated December 2015) were downloaded as reference data sets. Next, the number of raw reads for each sequencing file was counted.

Furthermore, this step provides information if sequencing depth of files was comparable. Databases were indexed to accelerate the mapping process and read mapping was performed using the program BWA. This algorithm does not perform global alignments but searches for base pair matches long enough to identify a single transcript. Resulting mapped reads were recorded and transcript names, lengths and read counts were reported. Finally, reads were normalized to TPMs (transcripts per million) to eliminate biases introduced by transcript length and sequencing depth. Gene and transcript lengths provided by the database versions of Ensembl and circBase were used for calculation of TPM values. This step allows a direct comparison of transcript quantities between the two different patient groups C and HR.

$$\text{reads per kilobase (RPK)} = \frac{\text{read counts of a transcript}}{\text{transcript length [bp]}}$$

$$\text{TPM} = \frac{\text{RPK}}{\Sigma\ \text{RPKs} \times 10^{-6}}$$

For analysis of differential gene expression of a transcript, $\lg_{10}$(fold change) values of expression levels were calculated and compared for pooled samples of C and HR patient group.

$$\log(\text{fold change}) = \frac{\text{patient group H [TPM]}}{\text{patient group C [TPM]}}$$

(log(fold change) > 0 for transcripts with a higher expression in patient group H,

log(fold change) = 0 without differences in gene expression in both patient groups,

and log(fold change) < 0 for transcripts with a lower expression in patient group H)
